# Non-uniqueness for the Transport Equation with Sobolev Vector Fields

**DOI:** 10.1007/s40818-018-0056-x

**Published:** 2018-12-07

**Authors:** Stefano Modena, László Székelyhidi

**Affiliations:** Institut für Mathematik, Universität Leipzig, 04109 Leipzig, Germany

**Keywords:** Transport equation, Renormalized solutions, Convex integration, Non-uniqueness, h-principle

## Abstract

We construct a large class of examples of non-uniqueness for the linear transport equation and the transport-diffusion equation with divergence-free vector fields in Sobolev spaces $W^{1, p}$.

## Introduction

This paper concerns the problem of (non)uniqueness of solutions to the transport equation in the periodic setting

1 $$\begin{aligned} \partial _t\rho +u\cdot \nabla \rho&=0, \end{aligned}$$

2 $$\begin{aligned} \rho _{|t=0}&=\rho ^0 \end{aligned}$$

where $\rho :[0,T]\times \mathbb {T}^d\rightarrow \mathbb {R}$ is a scalar density, $u:[0,T]\times \mathbb {T}^d\rightarrow \mathbb {R}^d$ is a given vector field and $\mathbb {T}^d = \mathbb {R}^d / \mathbb {Z}^d$ is the *d*-dimensional flat torus.

Unless otherwise specified, we assume in the following that $u \in L^1$ is *incompressible*, i.e.

3 $$\begin{aligned} \text {div }u=0 \end{aligned}$$

in the sense of distributions. Under this condition, (1) is formally equivalent to the continuity equation

4 $$\begin{aligned} \partial _t \rho + \text {div }(\rho u) = 0. \end{aligned}$$

It is well known that the theory of classical solutions to (1)–(2) is closely connected to the ordinary differential equation

5 $$\begin{aligned} \begin{aligned} \partial _t X(t,x)&=u(t, X(t,x)),\\ X(0,x)&= x, \end{aligned} \end{aligned}$$

via the formula $\rho (t, X(t,x))=\rho ^0(x)$. In particular, for Lipschitz vector fields *u* the well-posedness theory for (1)–(2) follows from the Cauchy–Lipschitz theory for ordinary differential equations applied to (5); on the other side, the inverse flow map $\Phi (t) := X(t)^{-1}$ solves the transport equation

6 $$\begin{aligned} \begin{aligned}&\partial _t \Phi + (u \cdot \nabla ) \Phi = 0, \\&\Phi |_{t=0} = \text {id}. \end{aligned} \end{aligned}$$

There are several PDE models, related, for instance, to fluid dynamics or to the theory of conservation laws (see for instance [19, 25, 33, 35, 36]), where one has to deal with vector fields which are not necessarily Lipschitz, but have lower regularity and therefore it is important to investigate the well-posedness of (1)–(2) in the case of non-smooth vector fields.

Starting with the groundbreaking work of DiPerna-Lions [26] there is a wealth of well-posedness results for vector fields which are Sobolev or BV (we refer to the recent survey [6], see also below) and in particular in recent years a lot of effort has been devoted to understanding how far the regularity assumptions can be relaxed. The main goal of this paper is to provide a lower bound on the regularity assumptions by showing, to our knowledge for the first time, that well-posedness can fail quite spectacularly even in the Sobolev setting, with $u \in C_t W^{1,{\tilde{p}}}_x := C([0,T]; W^{1,{\tilde{p}}}(\mathbb {T}^d))$ (see Theorem 1.2 for the precise statement). The mechanism we exploit to produce such “failure of uniqueness” is so strong that it can be applied also to the transport-diffusion equation

7 $$\begin{aligned} \partial _t \rho + \text {div }(\rho u) = \Delta \rho \end{aligned}$$

thus producing Sobolev vector fields $u \in C_t W^{1, {\tilde{p}}}_x$ for which uniqueness of solutions to (7)–(2) fails in the class of densities $\rho \in C_t L^p$ (see Theorem 1.9).

Both theorems can be generalized as follows: we can construct vector fields with arbitrary large regularity $u \in W^{{\tilde{m}},\tilde{p}}$, ${\tilde{m}} \in \mathbb {N}$, for which uniqueness of solutions to (1)–(2) or (7)–(2) fails, in the class of densities $\rho \in W^{m, p}$, with arbitrary large $m \in \mathbb {N}$; moreover, we can do that even when on the r.h.s. of (7) there is a higher order diffusion operator (see Theorems 1.6 and 1.10).

Before stating the precise statements of these results, we present a brief (and far from complete) overview of the main well-posedness achievements present in the literature. We start with the analysis of the well-posedness for the transport equation in class of bounded densities, then we pass to the analysis of well-posedness for the transport equation in the class of $L^p$-integrable densities, with the statement of our Theorems 1.2 and 1.6 and finally we discuss the transport-diffusion equation, with the statements of our Theorems 1.9 and 1.10. The last part of this introduction is devoted to a brief overview of the main techniques used in our proofs.

### The Case of Bounded Densities

The literature about *rough* vector fields mainly concerns the well-posedness of (1)–(2) in the class of bounded densities, $\rho \in L^\infty$. The reason for that can be found in the fact that the scientific community has been mainly interested in the well-posedness of ODE (5) and has used the PDE as a tool to attack the ODE problem: the general strategy is that a well-posedness result for the transport equation in the class of bounded densities yields a unique solution to the PDE (6) and thus one tries to prove that the flow $X(t) := \Phi (t)^{-1}$ is the unique meaningful solution, in the sense of *regular Lagrangian flow*, to the ODE (5). We refer to [6] for a precise definition of the notion of *regular Lagrangian flow* and for a detailed discussion about the link between the Eulerian and the Lagrangian approach.

Let us observe that for $\rho \in L^\infty$ the quantity $\rho u \in L^1$ and thus one can consider solutions to (1) (or, equivalently, to (4), since we are assuming incompressibility of the vector field) in distributional sense: $\rho$ is a *distributional or weak solution* if

8 $$\begin{aligned} \int _0^T \int _{\mathbb {T}^d} \rho [\partial _t \varphi + u \cdot \nabla \varphi ] dxdt= 0, \end{aligned}$$

for every $\varphi \in C^\infty _c ((0,T) \times \mathbb {T}^d)$. It is usually not difficult to prove existence of weak solutions, even if the vector field is very rough, taking advantage of the linearity of the equation. A much bigger issue is the uniqueness problem.

The first result in this direction dates back to DiPerna and Lions [26], when they proved uniqueness, in the class of bounded densities, for vector fields $u \in L^1_t W^{1,1}_x$ with bounded divergence. This result was extended in 2004 by Ambrosio [5] to vector fields $u \in L^1_t BV_x \cap L^\infty$ and with bounded divergence (see also [16, 17]) and very recently by Bianchini and Bonicatto [8] for vector fields $u \in L^1_t BV_x$ which are merely *nearly incompressible* (see, for instance, [6] for a definition of *nearly incompressibility*).

The proofs of these results are very subtle and involves several deep ideas and sophisticated techniques. We could however try to summarize the heuristic behind all of them as follows: (very) roughly speaking, a Sobolev or BV vector field *u* is Lipschitz-like (i.e. *Du* is bounded) on a large set and there is just a small “bad” set, where *Du* is very large. On the big set where *u* is “Lipschitz-like”, the classical uniqueness theory applies. Non-uniqueness phenomena could thus occur only on the small “bad” set. Uniqueness of solutions in the class of bounded densities is then a consequence of the fact that a *bounded* density $\rho$ can not “see” this bad set, or, in other words, cannot concentrate on this bad set.

With this rough heuristic in mind it is also perhaps not surprising that the theory cited above is heavily measure-theoretic. Nevertheless, the well-posedness for the ODE (5) fundamentally relies on the analysis and well-posedness theory of the associated PDE (1). More precisely in DiPerna and Lions [26], introduced the notion of *renormalized solution*. One calls a density $\rho \in L^1_{tx}$ renormalized for (1) (for given *u*), if for any $\beta \in L^{\infty }(\mathbb {R})\cap C^1(\mathbb {R})$ it holds

9 $$\begin{aligned} \partial _t\beta (\rho )+u\cdot \nabla \beta (\rho )=0 \end{aligned}$$

in the sense of distributions. Analogously to entropy-conditions for hyperbolic conservation laws, (9) provides additional stability under weak convergence. Key to the well-posedness theory is then showing that any bounded distributional solution $\rho$ of (1) is renormalized—this is done by showing convergence of the commutator

10 $$\begin{aligned} (u\cdot \nabla \rho )_{\varepsilon }-u_{\varepsilon }\cdot \nabla \rho _\varepsilon \,\rightarrow 0 \end{aligned}$$

arising from suitable regularizations.

As we mentioned, uniqueness at the PDE level in the class of bounded densities implies, in all the cases considered above, uniqueness at the ODE level (again in the sense of the *regular Lagrangian flow*). On the other hand, based on a self-similar mixing example of Aizenmann [1], Depauw [24] constructed an example of non-uniqueness for weak solutions with $\rho \in L^{\infty }((0,T)\times \mathbb {T}^d)$ and $u\in L^1(\varepsilon ,1;BV(\mathbb {T}^d))$ for any $\varepsilon >0$ but $u\notin L^1(0,1;BV(\mathbb {T}^d))$. This example has been revisited in [3, 4, 17, 37]. It should be observed, though, that the phenomenon of non-uniqueness in such “mixing” examples is *Lagrangian* in the sense that it is a consequence of the degeneration of the flow map *X*(*t*, *x*) as $t\rightarrow 0$; in particular, once again, the link between (1) and (5) is crucial.

### The Case of Unbounded Densities

There are important mathematical models, related, for instance, to the Boltzmann equation (see [25]), incompressible 2D Euler [19], or to the compressible Euler equations, in which the density under consideration is not bounded, but it belongs just to some $L^\infty _t L^p_x$ space. It is thus an important question to understand the well-posedness of the Cauchy problem (1)–(2) in such larger functional spaces.

As a first step, we observe that for a density $\rho \in L^\infty _t L^p_x$ and a field $u \in L^1_t L^1_x$, the product $\rho u$ is not well defined in $L^1$ and thus the notion of weak solution as in (8) has to be modified. There are several possibilities to overcome this issue. We mention two of them: either we require that $u \in L^1_t L^{p'}_x$, where $p'$ is the dual Hölder exponent to *p*, or we consider a notion of solution which cut off the regions where $\rho$ is unbounded. Indeed, this second possibility is encoded in the notion of renormalized solution (9).

The well-posedness theory provided by (9) for bounded densities is sufficient for the existence of a regular Lagrangian flow, which in turn leads to *existence* also for unbounded densities. For the *uniqueness*, an additional integrability condition is required:

#### Theorem 1.1

(DiPerna–Lions [26]) Let $p,{\tilde{p}}\in [1,\infty ]$ and let $u\in L^1(0,T;W^{1,\tilde{p}}(\mathbb {T}^d))$ be a vector field with $\text {div }u=0$. For any $\rho ^0\in L^p(\mathbb {T}^d)$ there exists a unique renormalized solution of (1)–(2), satisfying $\rho \in C([0,T];L^p(\mathbb {T}^d))$. Moreover, if

11 $$\begin{aligned} \frac{1}{p}+\frac{1}{{\tilde{p}}}\le 1 \end{aligned}$$

then this solution is *unique* among all weak solutions with $\rho \in L^{\infty }(0,T;L^p(\mathbb {T}^d))$.

As we have already observed for the case of bounded densities, also in this more general setting existence of weak and renormalized solutions is not a difficult problem. It is as well not hard to show uniqueness in the class of renormalized solutions, using the fact that renormalized solutions in $L^\infty _t L^p_x$ have constant in time $L^p$ norm (it suffices to choose $\beta$ as a bounded smooth approximation of $\tau \mapsto |\tau |^p$).

The crucial point in Theorem 1.1 concerns the uniqueness of the renormalized solution among all the weak solutions in $L^\infty _t L^p_x$, provided (11) is satisfied. The reason why such uniqueness holds can be explained by the same heuristic as in the case of bounded densities: a vector field in $W^{1,{\tilde{p}}}$ is “Lipschitz-like” except on a small bad set, which can not “be seen” by a density in $L^p$, if (11) holds, i.e. if *p*, although it is less than $\infty$, is sufficiently large w.r.t. ${\tilde{p}}$. On the more technical side, the integrability condition (11) is necessary in the proof in [26] to show convergence of the commutator (10) in $L^1_{loc}$.

The following question is therefore left open: does uniqueness of weak solutions hold in the class of densities $\rho \in L^\infty _t L^p_x$ for a vector field in $L^1_t L^{p'}_x \cap L^1_t W^{1, \tilde{p}}_x$, when (11) fails?

In a recent note Caravenna and Crippa [15], addressed this issue for the case $p=1$ and ${\tilde{p}}>1$, announcing the result that uniqueness holds under the additional assumption that *u* is continuous.

In this paper we show that if

12 $$\begin{aligned} \frac{1}{p} + \frac{1}{{\tilde{p}}} > 1 + \frac{1}{d-1}. \end{aligned}$$

then, in general, uniqueness fails. We remark that the Sobolev regularity of the vector field $u \in L^1_t W^{1,{\tilde{p}}}_x$ implies the existence of a unique regular Lagrangian flow (see in particular [7]). Nevertheless, quite surprisingly, our result shows that such Lagrangian uniqueness is of no help to get uniqueness on the Eulerian side.

Previously, examples of such Eulerian non-uniqueness have been constructed, for instance, in [18], based on the method of convex integration from [21], yielding merely bounded velocity *u* and density $\rho$. However, such examples do not satisfy the differentiability condition $u \in W^{1, {\tilde{p}}}$ for any ${\tilde{p}}\ge 1$ and therefore do not possess an associated Lagrangian flow.

Here is the statement of our first and main result.

#### Theorem 1.2

Let $$\varepsilon >0$$, $${\bar{\rho }} \in C^\infty ([0,T] \times \mathbb {T}^d)$$, with

$$\begin{aligned} \int _{\mathbb {T}^d} {\bar{\rho }}(0,x) dx = \int _{\mathbb {T}^d} {\bar{\rho }}(t,x) dx\, \text { for every }\, t \in [0,T]. \end{aligned}$$

Let $p \in (1, \infty )$, ${\tilde{p}} \in [1, \infty )$ such that (12) holds. Then there exist $\rho : [0,T] \times \mathbb {T}^d \rightarrow \mathbb {R}$, $u:[0,T] \times \mathbb {T}^d \rightarrow \mathbb {R}^d$ such that

- $$\rho \in C\bigl ([0,T]; L^p (\mathbb {T}^d)\bigr )$$, $$u \in C\bigl ([0,T]; W^{1,{\tilde{p}}} (\mathbb {T}^d)\cap L^{p'} (\mathbb {T}^d)\bigr )$$;
- $(\rho , u)$ is a weak solution to (1) and (3);
- at initial and final time $\rho$ coincides with ${\bar{\rho }}$, i.e.
  $$\begin{aligned} \rho (0,\cdot ) = {\bar{\rho }}(0, \cdot ), \quad \rho (T, \cdot ) = \bar{\rho }(T, \cdot ); \end{aligned}$$
- $\rho$ is $\varepsilon$-close to ${\bar{\rho }}$ i.e.
  $$\begin{aligned} \begin{aligned} \sup _{t \in [0,T]} \big \Vert \rho (t,\cdot ) - {\bar{\rho }}(t, \cdot )\big \Vert _{L^p(\mathbb {T}^d)}&\le \varepsilon . \end{aligned} \end{aligned}$$

Our theorem has the following immediate consequences.

#### Corollary 1.3

(Non-uniqueness) Assume (12). Let ${\bar{\rho }} \in C^\infty (\mathbb {T}^d)$ with $\int _{\mathbb {T}^d}{\bar{\rho }}\,dx=0$. Then there exist

$$\begin{aligned} \rho \in C\big ([0,T]; L^p(\mathbb {T}^d)\big ), \quad u \in C\big ([0,T]; W^{1,{\tilde{p}}} (\mathbb {T}^d)\cap L^{p'} (\mathbb {T}^d))\big ) \end{aligned}$$

such that $(\rho ,u)$ is a weak solution to (1), (3), and $\rho \equiv 0$ at $t=0$, $\rho \equiv {\bar{\rho }}$ at $t=T$.

#### Proof

Let $\chi : [0,T] \rightarrow \mathbb {R}$ such that $\chi \equiv 0$ on [0, *T* / 4], $\chi \equiv 1$ on [3*T* / 4, *T*]. Apply Theorem 1.2 with ${\bar{\rho }}(t,x) := \chi (t) {\bar{\rho }}(x)$. $\square$

#### Corollary 1.4

(Non-renormalized solution) Assume (12). Then there exist

$$\begin{aligned} \rho \in C\big ([0,T]; L^p(\mathbb {T}^d)\big ), \quad u \in C\big ([0,T]; W^{1,{\tilde{p}}} (\mathbb {T}^d)\cap L^{p'} (\mathbb {T}^d))\big ) \end{aligned}$$

such that $(\rho ,u)$ is a weak solution to (1), (3), and $\Vert \rho (t)\Vert _{L^p(\mathbb {T}^d)}$ is not constant in time.

#### Proof

Take a smooth map ${\bar{\rho }}(t,x)$ such that its spatial mean value is constant in time, but its $L^p$ norm is not constant in time. Apply Theorem 1.2 with such ${\bar{\rho }}$ and

$$\begin{aligned} \varepsilon := \frac{1}{4} \max _{t,s} \bigg | \Vert \rho (t)\Vert _{L^p(\mathbb {T}^d)} - \Vert \rho (s)\Vert _{L^p(\mathbb {T}^d)} \bigg |. \end{aligned}$$

$$\square $$

#### Remark 1.5

We list some remarks about the statement of the theorem.

1. Condition (12) implies that $d \ge 3$. In fact it is not clear if a similar statement could hold for $d=2$ - see for instance [2] for the case of autonomous vector fields.
2. Our theorem shows the optimality of the condition of DiPerna–Lions in (11), at least for sufficiently high dimension $d\ge 3$.
3. The requirement that ${\bar{\rho }}$ has constant (in time) spatial mean value is necessary because weak solutions to (1), (3) preserve the spatial mean.
4. The condition (12) implies that the $L^{p'}$-integrability of the velocity *u* does not follow from the Sobolev embedding theorem.
5. We expect that the statement of Theorem 1.2 remains valid if (12) is replaced by
  13 $$\begin{aligned} \frac{1}{p}+\frac{1}{{\tilde{p}}}>1+\frac{1}{d}. \end{aligned}$$
  It would be interesting to see if this condition is sharp in the sense that uniqueness holds provided
  $$\begin{aligned} \frac{1}{p} + \frac{1}{{\tilde{p}}} \le 1 + \frac{1}{d}\,. \end{aligned}$$
  In this regard we note that (13) implies $\tilde{p}<d$. Conversely, if $u\in W^{1,{\tilde{p}}}$ with ${\tilde{p}}>d$, the Sobolev embedding implies that *u* is continuous so that the uniqueness statement in [15] applies.
6. The given function ${\bar{\rho }}$ could be less regular than $C^\infty$, but we are not interested in following this direction here.
7. It can be shown that the dependence of $\rho ,u$ on time is actually $C^\infty _t$, not just continuous, since we treat time just as a parameter.

Inspired by the heuristic described above, the proof of our theorem is based on the construction of densities $\rho$ and vector fields *u* so that $\rho$ is, in some sense, concentrated on the “bad” set of *u*, provided (12) holds. To construct such densities and fields, we treat the linear transport equation (1) as a non-linear PDE, whose unknowns are both $\rho$ and *u*: this allows us to control the interplay between density and field. More precisely, we must deal with two opposite needs: on one side, to produce “anomalous” solutions, we need to highly concentrate $\rho$ and *u*; on the other side, too highly concentrated functions fail to be Sobolev or even $L^p$-integrable. The balance between these two needs is expressed by (12).

It is therefore possible to guess that, under a more restrictive assumption than (12), one could produce anomalous solutions enjoying much more regularity than just $\rho \in L^p$ and $u \in W^{1, {\tilde{p}}}$. Indeed, we can produce anomalous solutions as regular as we like, as shown in the next theorem, where (12) is replaced by (14).

#### Theorem 1.6

Let $$\varepsilon >0$$, $${\bar{\rho }} \in C^\infty ([0,T] \times \mathbb {T}^d)$$, with

$$\begin{aligned} \int _{\mathbb {T}^d} {\bar{\rho }}(0,x) dx = \int _{\mathbb {T}^d} {\bar{\rho }}(t,x) dx\, \text { for every }\, t \in [0,T]. \end{aligned}$$

Let $p, {\tilde{p}} \in [1, \infty )$ and $m, {\tilde{m}} \in \mathbb {N}$ such that

14 $$\begin{aligned} \frac{1}{p} + \frac{1}{{\tilde{p}}} > 1 + \frac{m + {\tilde{m}}}{d-1}. \end{aligned}$$

Then there exist $\rho : [0,T] \times \mathbb {T}^d \rightarrow \mathbb {R}$, $u:[0,T] \times \mathbb {T}^d \rightarrow \mathbb {R}^d$ such that

- $$\rho \in C([0,T], W^{m,p} (\mathbb {T}^d))$$, $$u \in C([0,T]; W^{{\tilde{m}},{\tilde{p}}} (\mathbb {T}^d))$$, $$\rho u \in C([0,1]; L^1 (\mathbb {T}^d))$$;
- $(\rho , u)$ is a weak solution to (1), (3);
- at initial and final time $\rho$ coincides with $\bar{\rho }$, i.e.
  $$\begin{aligned} \rho (0,\cdot ) = {\bar{\rho }}(0, \cdot ), \quad \rho (T, \cdot ) = \bar{\rho }(T, \cdot ); \end{aligned}$$
- $\rho$ is $\varepsilon$-close to ${\bar{\rho }}$ i.e.
  $$\begin{aligned} \begin{aligned} \sup _{t \in [0,T]} \big \Vert \rho (t,\cdot ) - {\bar{\rho }}(t, \cdot )\big \Vert _{W^{m,p}(\mathbb {T}^d)}&\le \varepsilon . \\ \end{aligned} \end{aligned}$$

#### Remark 1.7

The analogues of Corollaries 1.3 and 1.4 continue to hold in Theorems 1.6. Observe also that (14) reduces to (12) if we choose $m=0$ and ${\tilde{m}} =1$.

#### Remark 1.8

Contrary to Theorem 1.2, here we do not show that $u \in C([0,T], L^{p'}(\mathbb {T}^d))$. Here we prove that $\rho u \in C([0,T], L^1(\mathbb {T}^d))$ by showing that $\rho \in C([0,T]; L^s(\mathbb {T}^d))$ and $u \in C([0,T]; L^{s'}(\mathbb {T}^d))$ for some suitably chosen $s,s' \in (1, \infty )$. This is also the reason why in Theorem 1.6 we allow the case $p = 1$. Indeed, Theorem 1.2, for any given *p*, produces a vector field $u \in C_t L^{p'}_x$; on the contrary, Theorem 1.6 just produces a field $u \in C_t L^{s'}_x$, for some $s' < p'$.

### Extension to the Transport-Diffusion Equation

The mechanism of concentrating the density in the same set where the field is concentrated, used to construct anomalous solutions to the transport equation, can be used as well to prove non-uniqueness for the transport-diffusion equation (7).

The diffusion term $\Delta \rho$ “dissipates the energy” and therefore, heuristically, it helps for uniqueness. Non-uniqueness can thus be caused only by the transport term $\text {div }(\rho u)= u \cdot \nabla \rho$. Therefore, as a general principle, whenever a uniqueness result is available for the transport equation, the same result applies to the transport-diffusion equation (see, for instance, [19, 32, 34]). Moreover, the diffusion term $\Delta \rho$ is so strong that minimal assumptions on *u* are enough to have uniqueness: this is the case, for instance, if *u* is just bounded, or even $u \in L^r_t L^q_x$, with $2/r + d/q \le 1$ (see [31] and also [9], where this relation between *r*, *q*, *d* is proven to be sharp). Essentially, in this regime the transport term can be treated as a lower order perturbation of the heat equation.

On the other hand, the technique we use to prove non-uniqueness for the transport equation allows us to construct densities and fields, whose concentrations are so high that the transport term “wins” over the diffusion one and produces anomalous solutions to (7) as well. Roughly speaking, we have to compare $\text {div }(\rho u)$ with $\Delta \rho = \text {div }(\nabla \rho )$, or, equivalently, $\rho u$ with $\nabla \rho$, for instance in the $L^1$ norm. The way we construct concentration of $\rho$ and *u* can be arranged, under a more restrictive assumption than (12), so that

$$\begin{aligned} \Vert \rho u\Vert _{L^1} \approx 1, \qquad \Vert \nabla \rho \Vert _{L^1} \ll 1 \end{aligned}$$

[see the last inequality in (37) and (51)] and thus the transport term is “much larger” than the diffusion one. The precise statement is as follows.

#### Theorem 1.9

Let $$\varepsilon >0$$, $${\bar{\rho }} \in C^\infty ([0,T] \times \mathbb {T}^d)$$, with

$$\begin{aligned} \int _{\mathbb {T}^d} {\bar{\rho }}(0,x) dx = \int _{\mathbb {T}^d} {\bar{\rho }}(t,x) dx\, \text { for every }\, t \in [0,T]. \end{aligned}$$

Let $p \in (1, \infty )$, ${\tilde{p}} \in [1, \infty )$ such that

15 $$\begin{aligned} \frac{1}{p} + \frac{1}{{\tilde{p}}} > 1 + \frac{1}{d-1},\quad p' < d-1 \,. \end{aligned}$$

Then there exist $\rho : [0,T] \times \mathbb {T}^d \rightarrow \mathbb {R}$, $u:[0,T] \times \mathbb {T}^d \rightarrow \mathbb {R}^d$ such that

- $$\rho \in C\bigl ([0,T]; L^p (\mathbb {T}^d)\bigr )$$, $$u \in C\bigl ([0,T]; W^{1,{\tilde{p}}} (\mathbb {T}^d)\cap L^{p'} (\mathbb {T}^d)\bigr )$$;
- $(\rho , u)$ is a weak solution to (7) and (3);
- at initial and final time $\rho$ coincides with ${\bar{\rho }}$, i.e.
  $$\begin{aligned} \rho (0,\cdot ) = {\bar{\rho }}(0, \cdot ), \quad \rho (T, \cdot ) = \bar{\rho }(T, \cdot ); \end{aligned}$$
- $\rho$ is $\varepsilon$-close to ${\bar{\rho }}$ i.e.
  $$\begin{aligned} \begin{aligned} \sup _{t \in [0,T]} \big \Vert \rho (t,\cdot ) - {\bar{\rho }}(t, \cdot )\big \Vert _{L^p(\mathbb {T}^d)}&\le \varepsilon . \end{aligned} \end{aligned}$$

As for the transport equation, also for (7) we can generalize Theorem 1.9, to get densities and fields with arbitrary large regularity. Moreover, we can cover also the case of diffusion operators of arbitrary large order:

16 $$\begin{aligned} \partial _t \rho + \text {div }(\rho u) = L \rho , \end{aligned}$$

where *L* is a constant coefficient differential operator of order $k \in \mathbb {N}$, $k \ge 2$, not necessarily elliptic.

#### Theorem 1.10

Let $$\varepsilon >0$$, $${\bar{\rho }} \in C^\infty ([0,T] \times \mathbb {T}^d)$$, with

$$\begin{aligned} \int _{\mathbb {T}^d} {\bar{\rho }}(0,x) dx = \int _{\mathbb {T}^d} {\bar{\rho }}(t,x) dx\, \text { for every }\, t \in [0,T]. \end{aligned}$$

Let $p, {\tilde{p}} \in [1, \infty )$ and $m, {\tilde{m}} \in \mathbb {N}$ such that

17 $$\begin{aligned} \frac{1}{p} + \frac{1}{{\tilde{p}}} > 1 + \frac{m+{\tilde{m}}}{d-1},\quad {\tilde{p}} < \frac{d-1}{{\tilde{m}} + k -1}\,. \end{aligned}$$

Then there exist $\rho : [0,T] \times \mathbb {T}^d \rightarrow \mathbb {R}$, $u:[0,T] \times \mathbb {T}^d \rightarrow \mathbb {R}^d$ such that

- $$\rho \in C\bigl ([0,T]; W^{m,p} (\mathbb {T}^d)\bigr )$$, $$u \in C\bigl ([0,T]; W^{{\tilde{m}},{\tilde{p}}} (\mathbb {T}^d)\bigr )$$, $$\rho u \in C([0,1]; L^1(\mathbb {T}^d))$$;
- $(\rho , u)$ is a weak solution to (16) and (3);
- at initial and final time $\rho$ coincides with ${\bar{\rho }}$, i.e.
  $$\begin{aligned} \rho (0,\cdot ) = {\bar{\rho }}(0, \cdot ), \quad \rho (T, \cdot ) = \bar{\rho }(T, \cdot ); \end{aligned}$$
- $\rho$ is $\varepsilon$-close to ${\bar{\rho }}$ i.e.
  $$\begin{aligned} \begin{aligned} \sup _{t \in [0,T]} \big \Vert \rho (t,\cdot ) - {\bar{\rho }}(t, \cdot )\big \Vert _{W^{m,p}(\mathbb {T}^d)}&\le \varepsilon . \end{aligned} \end{aligned}$$

#### Remark 1.11

The analogues of Corollaries 1.3 and 1.4 continue to hold in Theorems 1.9 and 1.10. Remark 1.8 applies also to the statement of Theorem 1.10.

Observe also that, if we choose $m=0$, ${\tilde{m}}=1$, $k=2$, the first condition in (15) reduces to the first condition in (17), nevertheless (15) is not equivalent to (17). Indeed, (15) implies (17), but the viceversa is not true, in general. This can be explained by the fact that Theorem 1.9, for any given *p*, produces a vector field $u \in C_t L^{p'}_x$, while Theorem 1.10 just produces a field $u \in C_t L^{s'}_x$ for some $s' < p'$.

### Strategy of the Proof

Our strategy is based on the technique of convex integration that has been developed in the past years for the incompressible Euler equations in connection with Onsager’s conjecture, see [10–13, 22, 23, 29] and in particular inspired by the recent extension of the techniques to weak solutions of the Navier–Stokes equations in [14]. Whilst the techniques that led to progress and eventual resolution of Onsager’s conjecture in [29] are suitable for producing examples with Hölder continuous velocity (with small exponent) [30], being able to ensure that the velocity is in a Sobolev space $W^{1,{\tilde{p}}}$, i.e. with one full derivative, requires new ideas.

A similar issue appears when one wants to control the dissipative term $-\Delta u$ in the Navier–Stokes equations. Inspired by the theory of intermittency in hydrodynamic turbulence, Buckmaster and Vicol [14] introduced “intermittent Beltrami flows”, which are spatially inhomogeneous versions of the classical Beltrami flows used in [10–12, 22, 23]. In contrast to the homogeneous case, these have different scaling for different $L^q$ norms at the expense of a diffuse Fourier support. In particular, one can ensure small $L^q$ norm for small $q>1$, which in turn leads to control of the dissipative term.

In this paper we introduce concentrations to the convex integration scheme in a different way, closer in spirit to the $\beta$-model, introduced by Frisch et al. [27, 28] as a simple model for intermittency in turbulent flows. In addition to a large parameter $\lambda$ that controls the frequency of oscillations, we introduce a second large parameter $\mu$ aimed at controlling concentrations. Rather than working in Fourier space, we work entirely in *x*-space and use “Mikado flows”, introduced in [20] and used in [13, 29] as the basic building blocks. These building blocks consist of pairwise disjoint (periodic) pipes in which the divergence-free velocity and, in our case, the density are supported. In particular, our construction only works for dimensions $d\ge 3$. The oscillation parameter $\lambda$ controls the frequency of the periodic arrangement - the pipes are arranged periodically with period $1{/}\lambda$. The concentration parameter $\mu$ controls the relative (to $1{/}\lambda$) radius of the pipes and the size of the velocity and density. Thus, for large $\mu$ our building blocks consist of a $1{/}\lambda$-periodic arrangement of very thin pipes of total volume fraction $1{/}\mu ^{d-1}$ where the velocity and density are concentrated—see Proposition 4.1 and Remark 4.2 below.

We prove in details only Theorem 1.2, in Sects. 2–6. The proofs of Theorems 1.6, 1.9, 1.10 can be obtained from the one of Theorem 1.2 with minor changes. A sketch is provided in Sect. 7.

## Technical Tools

We start by fixing some notation:

- $\mathbb {T}^d = \mathbb {R}^d / \mathbb {Z}^d$ is the *d*-dimensional flat torus.
- For $p \in [1,\infty ]$ we will always denote by $p'$ its dual exponent.
- If *f*(*t*, *x*) is a smooth function of $t \in [0,T]$ and $x \in \mathbb {T}^d$, we denote by
  - $\Vert f\Vert _{C^k}$ the sup norm of *f* together with the sup norm of all its derivatives in time and space up to order *k*;
  - $\Vert f(t, \cdot )\Vert _{C^k(\mathbb {T}^d)}$ the sup norm of *f* together with the sup norm of all its spatial derivatives up to order *k* at fixed time *t*;
  - $\Vert f(t,\cdot )\Vert _{L^p(\mathbb {T}^d)}$ the $L^p$ norm of *f* in the spatial derivatives, at fixed time *t*. Since we will take always $L^p$ norms in the spatial variable (and never in the time variable), we will also use the shorter notation $\Vert f(t, \cdot )\Vert _{L^p} = \Vert f(t)\Vert _{L^p}$ to denote the $L^p$ norm of *f* in the spatial variable.
- $C^\infty _0(\mathbb {T}^d)$ is the set of smooth functions on the torus with zero mean value.
- $$\mathbb {N}= \{0,1,2, \dots \}$$.
- We will use the notation $C(A_1, \dots , A_n)$ to denote a constant which depends only on the numbers $A_1, \dots , A_n$.

We now introduce three technical tools, namely an improved Hölder inequality, an antidivergence operator and a lemma about the mean value of fast oscillating functions. These tools will be frequently used in the following. For a function $g \in C^\infty (\mathbb {T}^d)$ and $\lambda \in \mathbb {N}$, we denote by $g_\lambda : \mathbb {T}^d \rightarrow \mathbb {R}$ the $1{/}\lambda$ periodic function defined by

18 $$\begin{aligned} g_\lambda (x) := g(\lambda x). \end{aligned}$$

Notice that for every $k \in \mathbb {N}$ and $p \in [1, \infty ]$

$$\begin{aligned} \Vert D^k g_\lambda \Vert _{L^p(\mathbb {T}^d)} = \lambda ^k \Vert D^k g\Vert _{L^p(\mathbb {T}^d)}. \end{aligned}$$

### Improved Hölder Inequality

We start with the statement of the improved Hölder inequality, inspired by Lemma 3.7 in [14].

#### Lemma 2.1

Let $\lambda \in \mathbb {N}$ and $f,g: \mathbb {T}^d \rightarrow \mathbb {R}$ be smooth functions. Then for every $p \in [1, \infty ]$,

19 $$\begin{aligned} \bigg |\Vert fg_\lambda \Vert _{L^p} - \Vert f\Vert _{L^p} \Vert g\Vert _{L^p} \bigg | \le \frac{C_p}{\lambda ^{1/p}} \Vert f\Vert _{C^1} \Vert g\Vert _{L^p}, \end{aligned}$$

where all the norms are taken on $\mathbb {T}^d$. In particular

20 $$\begin{aligned} \Vert fg_\lambda \Vert _{L^p} \le \Vert f\Vert _{L^p} \Vert g\Vert _{L^p} + \frac{C_p}{\lambda ^{1/p}} \Vert f\Vert _{C^1} \Vert g\Vert _{L^p}. \end{aligned}$$

#### Proof

Let us divide $\mathbb {T}^d$ into $\lambda ^d$ small cubes $\{Q_j\}_j$ of edge $1{/}\lambda$. On each $Q_j$ we have Summing over *j* we getLet us now estimate the second term in the r.h.s. For $x,y \in Q_j$ it holds

$$\begin{aligned} \begin{aligned} \Big | |f(x)|^p - |f(y)|^p \Big |&\le \frac{C_p}{\lambda } \Vert f\Vert ^{p-1}_{C^0(\mathbb {T}^d)} \Vert \nabla f\Vert _{C^0(\mathbb {T}^d)} \le \frac{C_p}{\lambda } \Vert f\Vert ^p_{C^1(\mathbb {T}^d)}. \end{aligned} \end{aligned}$$

Thereforefrom which we get

$$\begin{aligned} \bigg |\Vert fg_\lambda \Vert ^p_{L^p} - \Vert f\Vert ^p_{L^p} \Vert g\Vert ^p_{L^p} \bigg | \le \frac{C_p}{\lambda } \Vert f\Vert ^p_{C^1} \Vert g\Vert ^p_{L^p}. \end{aligned}$$

Inequality (19) is now obtained by taking the $1{/}p$ power in the last formula and using that for $A,B > 0$, $|A - B|^p \le ||A|^p - |B|^p|$. Finally, the improved Hölder inequality (20) is an immediate consequence of (19). $\square$

### Antidivergence Operators

For $f \in C^\infty _0(\mathbb {T}^d)$ there exists a unique $u \in C^\infty _0(\mathbb {T}^d)$ such that $\Delta u = f$. The operator $\Delta ^{-1}: C^\infty _0(\mathbb {T}^d) \rightarrow C^\infty _0(\mathbb {T}^d)$ is thus well defined. We define the *standard antidivergence operator* as $\nabla \Delta ^{-1}: C^\infty _0(\mathbb {T}^d) \rightarrow C^\infty (\mathbb {T}^d; \mathbb {R}^d)$. It clearly satisfies $\text {div }(\nabla \Delta ^{-1} f) = f$.

#### Lemma 2.2

For every $k \in \mathbb {N}$ and $p \in [1, \infty ]$, the *standard* antidivergence operator satisfies the bounds

21 $$\begin{aligned} \big \Vert D^k (\nabla \Delta ^{-1} g) \big \Vert _{L^p} \le C_{k,p} \Vert D^k g\Vert _{L^p}. \end{aligned}$$

Moreover for every $\lambda \in \mathbb {N}$ it holds

22 $$\begin{aligned} \big \Vert D^k (\nabla \Delta ^{-1} g_\lambda )\big \Vert _{L^p} \le C_{k,p}\lambda ^{k-1} \Vert D^k g\Vert _{L^p}. \end{aligned}$$

#### Proof

For $p \in (1, \infty )$ from the Calderon-Zygmund inequality we get

23 $$\begin{aligned} \Vert D^k (\nabla \Delta ^{-1} g)\Vert _{W^{1,p}(\mathbb {T}^d)} \le C_{k,p} \Vert D^k g\Vert _{L^p(\mathbb {T}^d)}, \end{aligned}$$

from which (21) follows. For $p = \infty$, we use Sobolev embeddings to get

$$\begin{aligned} \begin{aligned} \Vert D^k (\nabla \Delta ^{-1} g)\Vert _{L^\infty (\mathbb {T}^d)}&\le C \Vert D^k (\nabla \Delta ^{-1} g)\Vert _{W^{1, d+1}(\mathbb {T}^d)} \\ \text {(by} \, (23)\, \text {with}\, p=d+1)&\le C_{k, d+1} \Vert D^k g\Vert _{L^{d+1}(\mathbb {T}^d)} \\&\le C_{k, \infty } \Vert D^k g\Vert _{L^{\infty }(\mathbb {T}^d)}. \end{aligned} \end{aligned}$$

For $p=1$ we use the dual characterization of $L^1$ norm. For every $f \in L^1$,

$$\begin{aligned} \begin{aligned} \Vert f\Vert _{L^1(\mathbb {T}^d)}&= \max \bigg \{ \int _{\mathbb {T}^d} f \varphi \ : \ \varphi \in L^\infty (\mathbb {T}^d), \ \Vert \varphi \Vert _{L^\infty (\mathbb {T}^d)} = 1 \bigg \} \\&= \sup \bigg \{ \int _{\mathbb {T}^d} f \varphi \ : \ \varphi \in C^\infty (\mathbb {T}^d), \ \Vert \varphi \Vert _{L^\infty (\mathbb {T}^d)} = 1 \bigg \}. \end{aligned} \end{aligned}$$

Moreover, if , it also holds

$$\begin{aligned} \Vert f\Vert _{L^1(\mathbb {T}^d)} = \sup \bigg \{ \int _{\mathbb {T}^d} f \varphi \ : \ \varphi \in C^\infty _0(\mathbb {T}^d), \ \Vert \varphi \Vert _{L^\infty (\mathbb {T}^d)} = 1 \bigg \}. \end{aligned}$$

Therefore (in the following formula $\partial ^k$ denotes any partial derivative of order *k*):

$$\begin{aligned} \begin{aligned} \Vert \partial ^k (\nabla \Delta ^{-1} g)\Vert _{L^1(\mathbb {T}^d)}&= \sup _{\begin{array}{c} \varphi \in C^\infty _0(\mathbb {T}^d) \\ \Vert \varphi \Vert _{L^\infty } = 1 \end{array}} \int _{\mathbb {T}^d} \partial ^k (\nabla \Delta ^{-1} g) \ \varphi \ dx \\&= \sup _{\begin{array}{c} \varphi \in C^\infty _0(\mathbb {T}^d) \\ \Vert \varphi \Vert _{L^\infty } = 1 \end{array}} \int _{\mathbb {T}^d} \partial ^k g \ \nabla \Delta ^{-1} \varphi \ dx \\ \text {(by H}\ddot{\mathrm{o}}\text {lder)}&\le \sup _{\begin{array}{c} \varphi \in C^\infty _0(\mathbb {T}^d) \\ \Vert \varphi \Vert _{L^\infty } = 1 \end{array}} \Vert \partial ^k g \Vert _{L^1(\mathbb {T}^d)} \Vert \nabla \Delta ^{-1} \varphi \Vert _{L^\infty (\mathbb {T}^d)} \\ \text {(using}\, (21)\, \text {with}\, p=\infty )&\le C_{0, \infty } \Vert \partial ^k g \Vert _{L^1(\mathbb {T}^d)} \sup _{\begin{array}{c} \varphi \in C^\infty _0(\mathbb {T}^d) \\ \Vert \varphi \Vert _{L^\infty } = 1 \end{array}} \Vert \varphi \Vert _{L^\infty (\mathbb {T}^d)} \\&\le C_{0, \infty } \Vert \partial ^k g \Vert _{L^1(\mathbb {T}^d)}, \end{aligned} \end{aligned}$$

from with (21) with $p=1$ follows. To prove (22), observe that

$$\begin{aligned} \nabla \Delta ^{-1} g_\lambda (x) = \frac{1}{\lambda } (\nabla \Delta ^{-1} g) (\lambda x). \end{aligned}$$

Therefore

$$\begin{aligned} \begin{aligned} \Vert D^k (\nabla \Delta ^{-1} g_\lambda )\Vert _{L^p(\mathbb {T}^d)}&\le \lambda ^{k-1} \Vert (D^k (\nabla \Delta ^{-1} g))(\lambda \ \cdot )\Vert _{L^p(\mathbb {T}^d)} \\&\le \lambda ^{k-1} \Vert D^k (\nabla \Delta ^{-1} g)\Vert _{L^p(\mathbb {T}^d)} \\ \text {(by}\, (21))&\le C_{k,p}\lambda ^{k-1} \Vert D^k g\Vert _{L^p(\mathbb {T}^d)}, \end{aligned} \end{aligned}$$

thus proving (22). $$\square $$

With the help of the standard antidivergence operator, we now define an *improved* antidivergence operator, which lets us gain a factor $\lambda ^{-1}$ when applied to functions of the form $f(x) g(\lambda x)$.

#### Lemma 2.3

Let $\lambda \in \mathbb {N}$ and $f,g: \mathbb {T}^d \rightarrow \mathbb {R}$ be smooth functions withThen there exists a smooth vector field $u : \mathbb {T}^d \rightarrow \mathbb {R}^d$ such that $\text {div }u = f g_\lambda$ and for every $k \in \mathbb {N}$ and $p \in [1,\infty ]$,

24 $$\begin{aligned} \Vert D^k u\Vert _{L^p} \le C_{k,p} \lambda ^{k-1} \Vert f\Vert _{C^{k+1}} \Vert g\Vert _{W^{k,p}}. \end{aligned}$$

We will write

$$\begin{aligned} u = {\mathcal {R}} (f g_\lambda ). \end{aligned}$$

#### Remark 2.4

The same result holds if *f*, *g* are vector fields and we want to solve the equation $\text {div }u = f \cdot g_\lambda$, where $\cdot$ denotes the scalar product.

#### Proof

Set

$$\begin{aligned} u := f \nabla \Delta ^{-1} g_\lambda - \nabla \Delta ^{-1} \Big (\nabla f \cdot \nabla \Delta ^{-1}g_\lambda \Big ). \end{aligned}$$

It is immediate from the definition that $\text {div }u = fg_\lambda$. We show that (24) holds for $k=0,1$. The general case $k \in \mathbb {N}$ can be easily proven by induction. It holds (the constant $C_{0,p}$ can change its value from line to line)

$$\begin{aligned} \begin{aligned} \Vert u\Vert _{L^p}&\le \Vert f\Vert _{C^0} \Vert \nabla \Delta ^{-1}g_\lambda \Vert _{L^p} + \big \Vert \nabla \Delta ^{-1} \big (\nabla f \cdot \nabla \Delta ^{-1} g_\lambda \big ) \big \Vert _{L^p} \\ \text {(by}\, (21))&\le \Vert f\Vert _{C^0} \Vert \nabla \Delta ^{-1}g_\lambda \Vert _{L^p} + C_{0,p} \Vert \nabla f\Vert _{C^0} \Vert \nabla \Delta ^{-1} g_\lambda \Vert _{L^p} \\ \text {(by}\, (22))&\le \frac{C_{0,p}}{\lambda } \Vert f\Vert _{C^1} \Vert g\Vert _{L^p}, \end{aligned} \end{aligned}$$

so that (24) holds for $k=0$. For $k=1$ we compute

$$\begin{aligned} \partial _j u = \partial _j f \nabla \Delta ^{-1} g_\lambda + f \partial _j \nabla \Delta ^{-1} g_\lambda - \nabla \Delta ^{-1} \Big ( \nabla \partial _j f \cdot \nabla \Delta ^{-1} g_\lambda + \nabla f \cdot \partial _j \nabla \Delta ^{-1} g_\lambda \Big ). \end{aligned}$$

Therefore, using again (21) and (22), (the constant $C_{1,p}$ can change its value from line to line)

$$\begin{aligned} \begin{aligned} \Vert \partial _j u\Vert _{L^p}&\le C_{1,p} \bigg [ \Vert \partial _j f\Vert _{C^0} \Vert \nabla \Delta ^{-1} g_\lambda \Vert _{L^p} + \Vert f\Vert _{C^0} \Vert \partial _j \nabla \Delta ^{-1} g_\lambda \Vert _{L^p} \\&\quad + \Vert \nabla \partial _j f\Vert _{C^0} \Vert \nabla \Delta ^{-1} g_\lambda \Vert _{L^p} + \Vert \nabla f\Vert _{C^0} \Vert \partial _j\nabla \Delta ^{-1} g_\lambda \Vert _{L^p} \bigg ]\\&\le C_{1,p} \bigg [ \frac{1}{\lambda } \Vert f\Vert _{C^1} \Vert g\Vert _{L^p} + \Vert f\Vert _{C^0} \Vert \partial _j g\Vert _{L^p} \\&\quad + \frac{1}{\lambda } \Vert f\Vert _{C^2} \Vert g\Vert _{L^p} + \Vert f\Vert _{C^1} \Vert \partial _j g\Vert _{L^p} \bigg ] \\&\le C_{1,p} \bigg [ \Vert f\Vert _{C^1} \Vert \partial _j g\Vert _{L^p} + \frac{1}{\lambda } \Vert f\Vert _{C^2} \Vert g\Vert _{L^p} \bigg ] \\&\le C_{1,p} \Vert f\Vert _{C^2} \Vert g\Vert _{W^{1,p}}. \end{aligned} \end{aligned}$$

$$\square $$

#### Remark 2.5

Assume *f* and *g* are smooth function of (*t*, *x*), $t \in [0,T]$, $x \in \mathbb {T}^d$. If at each time *t* they satisfy (in the space variable) the assumptions of Lemma 2.3, then we can apply ${\mathcal {R}}$ at each time and define

$$\begin{aligned} u(t, \cdot ) := {\mathcal {R}} \Big ( f(t, \cdot ) g_\lambda (t, \cdot ) \Big ), \end{aligned}$$

where $g_\lambda (t,x) = g(t, \lambda x)$. It follows from the definition of ${\mathcal {R}}$ that *u* is a smooth function of (*t*, *x*).

### Mean Value and Fast Oscillations

#### Lemma 2.6

Let $\lambda \in \mathbb {N}$ and $f,g: \mathbb {T}^d \rightarrow \mathbb {R}$ be smooth functions withThen


#### Proof

We divide $\mathbb {T}^d$ into small cubes $\{Q_j\}$ of edge $1/\lambda$. For each $Q_j$, choose a point $x_j \in Q_j$. We have

$$\begin{aligned} \begin{aligned} \bigg |\int _{\mathbb {T}^d} f(x) g(\lambda x) dx \bigg |&= \bigg | \sum _j \int _{Q_j} f(x) g(\lambda x) dx \bigg |\\&= \bigg |\sum _j \int _{Q_j} \big [ f(x) - f(x_j) \big ] g(\lambda x) dx \bigg | \\&\le \sum _j \int _{Q_j} \big | f(x) - f(x_j) \big | |g(\lambda x)| dx \\&\le \frac{\sqrt{d} \Vert f\Vert _{C^1}\Vert g\Vert _{L^1(\mathbb {T}^d)}}{\lambda }. \end{aligned} \end{aligned}$$

$$\square $$

## Statement of the Main Proposition and Proof of Theorem 1.2

We assume without loss of generality that $T=1$ and $\mathbb {T}^d$ is the periodic extension of the unit cube $[0,1]^d$. The following proposition contains the key facts used to prove Theorem 1.2. Let us first introduce the *continuity-defect* equation:

25 $$\begin{aligned} \left\{ \begin{aligned}&\partial _t \rho + \text {div }(\rho u) = -\, \text {div }R, \\&\text {div }u = 0. \end{aligned} \right. \end{aligned}$$

We will call *R* the *defect field*. For $\sigma >0$ set $I_\sigma = (\sigma , 1 - \sigma )$. Recall that we are assuming $p \in (1, \infty )$.

### Proposition 3.1

There exists a constant $M>0$ such that the following holds. Let $\eta , \delta , \sigma > 0$ and let $(\rho _0, u_0, R_0)$ be a smooth solution of the continuity-defect equation (25). Then there exists another smooth solution $(\rho _1, u_1, R_1)$ of (25) such that

26a $$\begin{aligned} \Vert \rho _1(t) - \rho _0(t)\Vert _{L^p(\mathbb {T}^d)}&\le {\left\{ \begin{array}{ll} M \eta \Vert R_0(t)\Vert ^{1/p}_{L^1(\mathbb {T}^d)}, &{} t \in I_{\sigma /2}, \\ 0, &{} t \in [0,1] {\setminus } I_{\sigma /2}, \end{array}\right. } \end{aligned}$$

26b $$\begin{aligned} \Vert u_1(t) - u_0(t)\Vert _{L^{p'}(\mathbb {T}^d)}&\le {\left\{ \begin{array}{ll} M \eta ^{-1} \Vert R_0(t)\Vert ^{1/p'}_{L^1(\mathbb {T}^d)}, &{} t \in I_{\sigma /2}, \\ 0, &{} t \in [0,1] {\setminus } I_{\sigma /2}, \end{array}\right. } \end{aligned}$$

26c $$\begin{aligned} \Vert u_1(t) - u_0(t)\Vert _{W^{1,{\tilde{p}}}(\mathbb {T}^d)}&\le \delta , \end{aligned}$$

26d $$\begin{aligned} \Vert R_1(t)\Vert _{L^1(\mathbb {T}^d)}&\le {\left\{ \begin{array}{ll} \delta , &{} t \in I_\sigma , \\ \Vert R_0(t)\Vert _{L^1(\mathbb {T}^d)} + \delta , &{} t \in I_{\sigma /2} {\setminus } I_\sigma , \\ \Vert R_0(t)\Vert _{L^1(\mathbb {T}^d)}, &{} t \in [0,1] {\setminus } I_{\sigma /2}. \end{array}\right. } \end{aligned}$$

### Proof of Theorem 1.2 assuming Proposition 3.1

Let *M* be the constant in Proposition 3.1. Let $\tilde{\varepsilon }>0$ and $\eta >0$ (their precise value will be fixed later, with $\eta$ depending on ${{\tilde{\varepsilon }}}$). Let $\sigma _q = \delta _q := 2^{-q}$ and $I_q := I_{\sigma _q} = (\sigma _q, 1 - \sigma _q)$.

We construct a sequence $(\rho _q, u_q, R_q)$ of solutions to (25) as follows. Let $\phi _0, \phi , \phi _1: [0,1] \rightarrow \mathbb {R}$ three smooth functions such that

$$\begin{aligned} \phi _0(t) + \phi (t) + \phi _1(t) = 1 \quad \text { for every }\, t \in [0,1], \end{aligned}$$

and

$$\begin{aligned} \begin{aligned} \phi _0(t) = 1&\text { on } [0,{\tilde{\varepsilon }}], \\ \phi (t) = 1&\text { on } [2{\tilde{\varepsilon }}, 1 - 2 {\tilde{\varepsilon }}], \\ \phi _1(t) = 1&\text { on } [1 - {\tilde{\varepsilon }}, 1]. \end{aligned} \end{aligned}$$

Set

27 $$\begin{aligned} \begin{aligned} \rho _0(t)&:= \phi _0(t) {\bar{\rho }}(0) + \phi (t) {\bar{\rho }}(t) + \phi _1(t) {\bar{\rho }}(1), \\ u_0(t)&:= 0, \\ R_0(t)&:= -\, \nabla \Delta ^{-1} \big ( \partial _t \rho _0(t) + \text {div }(\rho _0(t) u_0(t)) \big ) = -\, \nabla \Delta ^{-1} \big ( \partial _t \rho _0(t) \big ), \end{aligned} \end{aligned}$$

where the antidivergence is taken with respect to the spatial variable.

Assume now that $(\rho _q, u_q, R_q)$ is defined. Let $(\rho _{q+1}, u_{q+1}, R_{q+1})$ be the solution to the continuity-defect equation, which is obtained by applying Proposition 3.1 to $(\rho _q, u_q, R_q)$, $\eta$,

$$\begin{aligned} \delta = \delta _{q+2}, \quad \sigma = \sigma _{q+1} \quad \text {(and thus } \sigma /2 = \sigma _{q+2}). \end{aligned}$$

### Lemma 3.2

The following inductive estimates are satisfied: 
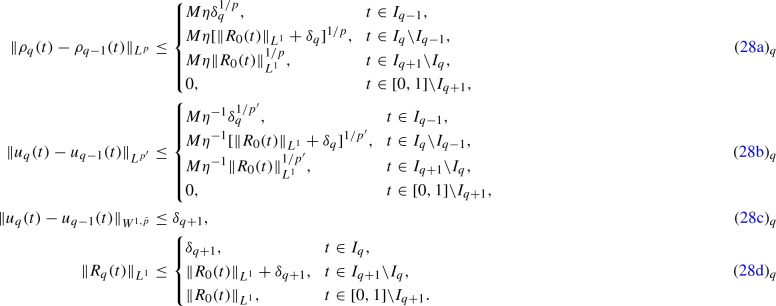


### Proof

For $q=0$, (28a)$_{q}$–(28c)$_{q}$ do not apply, whereas (28d)$_{q}$ is trivially satisfied, since $I_0 = \emptyset$. Assume now that (28a)$_{q}$–(28d)$_{q}$ hold and let us prove (28a)$_{q+1}$–(28d)$_{q+1}$. From (26a) we get

$$\begin{aligned} \Vert \rho _{q+1}(t) - \rho _q(t)\Vert _{L^p} \le {\left\{ \begin{array}{ll} M \eta \Vert R_q(t)\Vert _{L^1}^{1/p}, &{} t \in I_{q+2}, \\ 0, &{} t \in [0,1] setminus I_{q+2}. \end{array}\right. } \end{aligned}$$

Therefore, using the inductive assumption (28d)$_{q}$, we get:

- if $$t \in I_q$$,
  $$\begin{aligned} \Vert \rho _{q+1}(t) - \rho _q(t)\Vert _{L^p} \le M \eta \Vert R_q(t)\Vert _{L^1}^{1/p}, \le M \eta \delta _{q+1}^{1/p}; \end{aligned}$$
- if $$t \in I_{q+1} {{\setminus }} I_q$$,
  $$\begin{aligned} \Vert \rho _{q+1}(t) - \rho _q(t)\Vert _{L^p} \le M \eta \Vert R_q(t)\Vert _{L^1}^{1/p} \le M \eta \Big [|R_0(t)\Vert _{L^1} + \delta _{q+1} \Big ]^{1/p}; \end{aligned}$$
- if $$t \in I_{q+2} {{\setminus }} I_{q+1}$$,
  $$\begin{aligned} \Vert \rho _{q+1}(t) - \rho _q(t)\Vert _{L^p} \le M \eta \Vert R_q(t)\Vert _{L^1}^{1/p} \le M \eta \Vert R_0(t)\Vert _{L^1}^{1/p}, \end{aligned}$$

and thus (28a)$_{q+1}$ holds. Estimate (28b)$_{q+1}$ can be proven similarly. Estimate (28c)$_{q+1}$ is an immediate consequence of (26c). Finally, from (26d), we get

$$\begin{aligned} \Vert R_{q+1}(t)\Vert _{L^1} \le {\left\{ \begin{array}{ll} \delta _{q+2}, &{} t \in I_{q+1}, \\ \Vert R_q(t)\Vert _{L^1} + \delta _{q+2}, &{} t \in I_{q+2} {{\setminus }} I_{q+1}, \\ \Vert R_q(t)\Vert _{L^1}, &{} t \in [0,1] {{\setminus }} I_{q+2}. \end{array}\right. } \end{aligned}$$

Therefore, using the inductive assumption (28d)$_{q}$, we get:

- if $$t \in I_{q+2} {{\setminus }} I_{q+1}$$,
  $$\begin{aligned} \Vert R_{q+1}(t)\Vert _{L^1} \le \Vert R_q(t)\Vert _{L^1} + \delta _{q+2} \le \Vert R_0(t)\Vert _{L^1} + \delta _{q+2}; \end{aligned}$$
- if $$t \in [0,1] {{\setminus }} I_{q+2}$$,
  $$\begin{aligned} \Vert R_{q+1}(t)\Vert _{L^1} \le \Vert R_q(t)\Vert _{L^1} \le \Vert R_0(t)\Vert _{L^1}, \end{aligned}$$

from which (28d)$_{q+1}$ follows.

It is now an immediate consequence of the previous lemma that there exists

29 $$\begin{aligned} \rho \in C((0,1); L^p(\mathbb {T}^d)), \qquad u \in C((0,1); W^{1, \tilde{p}}(\mathbb {T}^d)) \cap C((0,1); L^{p'}(\mathbb {T}^d)) \end{aligned}$$

such that for every compact subset $K \subseteq (0,1)$

$$\begin{aligned}&\max _{t \in K} \Vert \rho _q(t) - \rho (t)\Vert _{L^p} \rightarrow 0 \\&\max _{t \in K} \Vert u_q(t) - u(t)\Vert _{L^{p'}} \rightarrow 0 \\&\max _{t \in K} \Vert u_q(t) - u(t)\Vert _{W^{1, {\tilde{p}}}} \rightarrow 0 \\&\max _{t \in K} \Vert R_q(t)\Vert _{L^1} \rightarrow 0, \end{aligned}$$

as $q \rightarrow \infty$, from which it follows that $\rho , u$ solves (3)–(4) [or (1)–(3)] in the sense of distributions. This proves part (b) of the statement.

We need now the following estimate. Let $t \in (0,1)$ and let $q^* = q^*(t) \in \mathbb {N}$ so that $t \in I_{q^*} {\setminus } I_{q^* -1}$. By the inductive estimate ((28a)),

30 $$\begin{aligned} \begin{aligned} \Vert \rho (t) - \rho _0(t)\Vert _{L^p}&\le \sum _{q=1}^\infty \Vert \rho _q(t) - \rho _{q-1}(t)\Vert _{L^p} \\&= \Vert \rho _{q^*-1}(t) - \rho _{q^*-2}(t)\Vert _{L^p} + \Vert \rho _{q^*}(t) - \rho _{q^*-1}(t)\Vert _{L^p} \\&\quad + \sum _{q=q^*+1}^\infty \Vert \rho _q(t) - \rho _{q-1}(t)\Vert _{L^p} \\&\le M \eta \Bigg [ \Vert R_0(t)\Vert ^{1/p}_{L^1} + \Big ( \Vert R_0(t)\Vert _{L^1} + \delta _{q^*}\Big ) ^{1{/}p} + \sum _{q=q^*+1}^\infty \delta _q^{1{/}p} \Bigg ]. \end{aligned} \end{aligned}$$

Let us now prove that $\Vert \rho (t) - {\bar{\rho }}(0)\Vert _{L^p} \rightarrow 0$ as $t \rightarrow 0$. Observe that, for $t < {\tilde{\varepsilon }}$, $\rho _0(t) = \bar{\rho }(0)$ and $R_0(t) = 0$. Hence, if $t < {\tilde{\varepsilon }}$,

$$\begin{aligned} \begin{aligned} \Vert \rho (t) - {\bar{\rho }}(0)\Vert _{L^p}&= \Vert \rho (t) - \rho _0(t)\Vert _{L^p} \\ \text {(by } (30))&\le M \eta \Bigg [ \Vert R_0(t)\Vert ^{1/p}_{L^1} + \Big ( \Vert R_0(t)\Vert _{L^1} + \delta _{q^*}\Big ) ^{1/p} + \sum _{q=q^*+1}^\infty \delta _q^{1{/}p} \Bigg ] \\&= M \eta \sum _{q=q^*}^\infty \delta _q^{1/p}, \end{aligned} \end{aligned}$$

and the conclusion follows observing that $q^* = q^*(t) \rightarrow \infty$ as $t \rightarrow 0$. In a similar way the limit $\Vert \rho (t) - \bar{\rho }(1)\Vert _{L^p} \rightarrow 0$ as $t \rightarrow 1$ can be shown. This completes the proof of parts (a) and (c) of the statement.

Let us now prove part (d). We first observe that, for the $\varepsilon$ given in the statement of the theorem, we can choose ${\tilde{\varepsilon }}$ small enough, so that for every $t \in [0,1]$,

31 $$\begin{aligned} \Vert \rho _0(t) - {\bar{\rho }}(t)\Vert _{L^p} \le \frac{\varepsilon }{2}. \end{aligned}$$

Indeed, if $t \in [2 {\tilde{\varepsilon }}, 1 - 2{\tilde{\varepsilon }}]$, then $\rho _0(t) = {\bar{\rho }}(t)$. If $t \in [0, 2{\tilde{\varepsilon }}] \cup [1 - 2 {\tilde{\varepsilon }}, 1]$, then

$$\begin{aligned} \begin{aligned} \Vert \rho _0(t) - {\bar{\rho }}(t)\Vert _{L^p}&\le |\phi _0(t)| \Vert \bar{\rho }(0) - {\bar{\rho }}(t)\Vert _{L^p} + |\phi _1(t)| \Vert {\bar{\rho }}(1) - \bar{\rho }(t)\Vert _{L^p} \le \frac{\varepsilon }{2}, \end{aligned} \end{aligned}$$

where the last inequality follows, by choosing ${\tilde{\varepsilon }}$ sufficiently small. Therefore, for every $t \in [0,1]$,

$$\begin{aligned} \begin{aligned} \Vert \rho (t) - {\bar{\rho }}(t)\Vert _{L^p}&\le \Vert \rho (t) - \rho _0(t)\Vert _{L^p} + \Vert \rho _0(t) - {\bar{\rho }}(t)\Vert _{L^p} \\ \text {(by } (30))&\le M \eta \Bigg [ \Vert R_0(t)\Vert ^{1/p}_{L^1} + \Big ( \Vert R_0(t)\Vert _{L^1} + \delta _{q^*}\Big ) ^{1/p} + \sum _{q=q^*+1}^\infty \delta _q^{1/p} \Bigg ] + \frac{\varepsilon }{2} \\&\le M \eta \max _{t \in [0,1]} \Bigg [\Vert R_0(t)\Vert ^{1/p}_{L^1} + \Big ( \Vert R_0(t)\Vert _{L^1} + 1 \Big ) ^{1/p} + \sum _{q=1}^\infty \delta _q^{1/p} \Bigg ] + \frac{\varepsilon }{2} \\&\le \varepsilon , \end{aligned} \end{aligned}$$

if $\eta$ is chosen small enough (depending on $R_0$ and thus on ${\tilde{\varepsilon }}$). This proves part (d) of the statement, thus concluding the proof of the theorem. $\square$

## The Perturbations

In this and the next two sections we prove Proposition 3.1. In particular in this section we fix the constant *M* in the statement of the proposition, we define the functions $\rho _1$ and $u_1$ and we prove some estimates on them. In Sect. 5 we define $R_1$ and we prove some estimates on it. In Sect. 6 we conclude the proof of Proposition 3.1, by proving estimates (26a)–(26d).

### Mikado Fields and Mikado Densities

The first step towards the definition of $\rho _1, u_1$ is the construction of *Mikado fields* and *Mikado densities*.

We start by fixing a function $\Phi \in C^\infty _c(\mathbb {R}^{d-1})$ such that

$$\begin{aligned} \mathrm {supp} \ \Phi \subseteq (0,1)^{d-1}, \quad \int _{\mathbb {R}^{d-1}} \Phi = 0, \quad \int _{\mathbb {R}^{d-1}} \Phi ^2 = 1. \end{aligned}$$

Let $\Phi _\mu (x) := \Phi (\mu x)$ for $\mu > 0$. Let $a \in \mathbb {R}$. For every $k \in \mathbb {N}$, it holds

32 $$\begin{aligned} \begin{aligned} \Vert D^k (\mu ^a \Phi _\mu )\Vert _{L^r(\mathbb {R}^{d-1})}&= \mu ^{a+k- (d-1)/r} \Vert D^k\Phi \Vert _{L^r(\mathbb {R}^{d-1})}. \\ \end{aligned} \end{aligned}$$

#### Proposition 4.1

Let $$a, b \in \mathbb {R}$$ with

33 $$\begin{aligned} a+b = d-1. \end{aligned}$$

For every $\mu >2d$ and $j=1, \dots , d$ there exist a *Mikado density* $\Theta _{\mu }^{j} : \mathbb {T}^d \rightarrow \mathbb {R}$ and a *Mikado field* $W_\mu ^j :\mathbb {T}^d \rightarrow \mathbb {R}^d$ with the following properties.

- It holds 34 where $\{e_j\}_{j=1,\dots ,d}$ is the standard basis in $\mathbb {R}^d$.
- For every $k \in \mathbb {N}$ and $r \in [1,\infty ]$
  35 $$\begin{aligned} \begin{aligned} \Vert D^k \Theta _\mu ^j\Vert _{L^r(\mathbb {T}^d)}&\le \Vert \Phi \Vert _{L^r(\mathbb {R}^{d-1})} \mu ^{a + k - (d-1) /r}, \\ \Vert D^k W_\mu ^j\Vert _{L^{r}(\mathbb {T}^d)}&\le \Vert \Phi \Vert _{L^r(\mathbb {R}^{d-1})} \mu ^{b + k - (d-1)/r}, \\ \end{aligned} \end{aligned}$$
- For $$j \ne k$$, $$\mathrm {supp} \ \Theta _\mu ^j = \mathrm {supp} \ W_\mu ^j$$ and $$\mathrm {supp} \ \Theta _\mu ^j \cap \mathrm {supp} \ W_{\mu }^k = \emptyset $$.

#### Remark 4.2

In particular notice that if we choose

$$\begin{aligned} a = \frac{d-1}{p}, \qquad b = \frac{d-1}{p'} \end{aligned}$$

and we define the constant *M* in the statement of Proposition 3.1 as

36 $$\begin{aligned} M := 2d \max \Big \{ \Vert \Phi \Vert _{L^\infty (\mathbb {R}^{d-1})}, \Vert \Phi \Vert ^2_{L^\infty (\mathbb {R}^{d-1})}, \Vert \nabla \Phi \Vert _{L^\infty (\mathbb {R}^{d-1})}\Big \}, \end{aligned}$$

then the following estimates hold:

37 $$\begin{aligned} \begin{aligned} \sum _{j=1}^d \Vert \Theta _\mu ^j\Vert _{L^p(\mathbb {T}^d)}, \ \sum _{j=1}^d \Vert W_\mu ^j\Vert _{L^{p'}(\mathbb {T}^d)}, \ \sum _{j=1}^d \Vert \Theta _\mu ^j W_\mu ^j\Vert _{L^1(\mathbb {T}^d)}&\le \frac{M}{2}, \\ \end{aligned} \end{aligned}$$

and

38 $$\begin{aligned} \begin{aligned} \Vert \Theta _\mu ^j\Vert _{L^1(\mathbb {T}^d)}, \ \Vert W_\mu ^j\Vert _{L^{1}(\mathbb {T}^d)}, \ \Vert W_\mu ^j\Vert _{W^{1, {\tilde{p}}}} \le M \mu ^{-\gamma }, \end{aligned} \end{aligned}$$

where

$$\begin{aligned} \gamma = \min \big \{ \gamma _1, \gamma _2, \gamma _3 \big \} > 0 \end{aligned}$$

and

$$\begin{aligned} \begin{aligned} \gamma _1&:= (d-1) \bigg (1 - \frac{1}{p} \bigg )> 0, \\ \gamma _2&:= (d-1) \bigg (1 - \frac{1}{p'} \bigg )> 0, \\ \gamma _3&:= -\, 1 - (d-1) \bigg [\frac{1}{p'} - \frac{1}{{\tilde{p}}} \bigg ] = (d-1) \bigg [\frac{1}{p} + \frac{1}{{\tilde{p}}} - \bigg ( 1 + \frac{1}{d-1} \bigg ) \bigg ] > 0. \end{aligned} \end{aligned}$$

Notice that $\gamma _3 >0$ by (12).

#### Proof of Proposition 4.1

*Step 1* For each $j=1,\dots , d$, we define the (non-periodic) Mikado density ${\tilde{\Theta }}_{\mu }^j : \mathbb {R}^d \rightarrow \mathbb {R}$

39a $$\begin{aligned} {\tilde{\Theta }}_\mu ^j(x_1, \dots , x_n) := \mu ^a \Phi _\mu (x_1, \dots , x_{j-1}, x_{j+1}, \dots , x_d) \end{aligned}$$

and the (non-periodic) Mikado field ${\tilde{W}}_\mu ^j : \mathbb {R}^d \rightarrow \mathbb {R}^d$

39b $$\begin{aligned} {\tilde{W}}_\mu ^j (x_1, \dots , x_n) := \mu ^b \Phi _\mu (x_1, \dots , x_{j-1}, x_{j+1}, \dots , x_d) e_j. \end{aligned}$$

Notice that for the non-periodic Mikado densities40where the last equality follows from (33). Moreover, from (32) we get

41 $$\begin{aligned} \begin{aligned} \Vert D^k {\tilde{\Theta }}_\mu ^j\Vert _{L^r((0,1)^d)}&= \mu ^{a + k - (d-1)/r}\Vert D^k\Phi \Vert _{L^r(\mathbb {R}^{d-1})} \\ \Vert D^k {\tilde{W}}_\mu ^j\Vert _{L^{r}((0,1)^d)}&= \mu ^{b + k - (d-1)/r}\Vert D^k\Phi \Vert _{L^{r}(\mathbb {R}^{d-1})} \\ \end{aligned} \end{aligned}$$

*Step 2* We define $\Theta _\mu ^j : \mathbb {T}^d \rightarrow \mathbb {R}$ and $W_\mu ^j :\mathbb {T}^d \rightarrow \mathbb {R}^d$ as the 1-periodic extension of $\tilde{\Theta }_\mu ^j$, ${\tilde{W}}_\mu ^j$ respectively. Such periodic extensions are well defined, since $\mathrm {supp} \ \Phi \subseteq (0,1)^{d-1}$ and ${\tilde{\Theta }}_\mu ^j$, ${\tilde{W}}_\mu ^j$ do not depend on the *j*-th coordinate. Equations (34) and estimates (35) come from the corresponding equations (40) and estimates (41) for the non-periodic Mikado densities and fields.

*Step 3* Finally notice that conditions (c) in the statement are not verified by $\Theta _\mu ^j$ and $W_\mu ^j$ defined in Step 2. However we can achieve (c), using that $\mu > 2d$ and redefining $\Theta _\mu ^j, W_\mu ^j$ after a suitable translation of the independent variable $x \in \mathbb {T}^d$ for each $j=1,\dots , d$. $\square$

### Definition of the Perturbations

We are now in a position to define $\rho _1$, $u_1$. The constant *M* has already been fixed in (36). Let thus $\eta , \delta , \sigma >0$ and $(\rho _0, u_0, R_0)$ be a smooth solution to the continuity-defect equation (25).

Let

$$\begin{aligned} \begin{aligned} \lambda \in \mathbb {N}&\text { ``oscillation''}, \\ \mu > 2d&\text { ``concentration''} \end{aligned} \end{aligned}$$

be two constant, which will be fixed in Sect. 6. Let $\psi \in C^\infty _c((0,1))$ such that $\psi \equiv 0$ on $[0,\sigma /2] \cup [1-\sigma /2, 1]$, $\psi \equiv 1$ on $[\sigma , 1 - \sigma ]$ and $|\psi | \le 1$. We denote by $R_{0,j}$ the components of $R_0$, i.e.

$$\begin{aligned} R_0(t,x) := \sum _{j=1}^d R_{0,j}(t,x) e_j. \end{aligned}$$

For $j=1,\dots , d$, let $\chi _j \in C^\infty ([0,1] \times \mathbb {T}^d)$ be such that

$$\begin{aligned} \chi _j(t,x) = {\left\{ \begin{array}{ll} 0, &{} \text {if } |R_{0,j}(t,x)| \le \delta /(4d), \\ 1, &{} \text {if } |R_{0,j}(t,x)| \ge \delta /(2d), \end{array}\right. } \end{aligned}$$

and $$|\chi _j| \le 1$$.

We set

$$\begin{aligned} \rho _1 := \rho _0 + \vartheta + \vartheta _c , \qquad u_1 := u_0 + w + w_c, \end{aligned}$$

where $\vartheta , \vartheta _c, w, w_c$ are defined as follows. First of all, let $\Theta _\mu ^j$, $W_\mu ^j$, $j=1,\dots , d$, be the Mikado densities and flows provided by Proposition 4.1, with *a*, *b* chosen as in Remark 4.2. We set42We will also use the shorter notation

$$\begin{aligned} \begin{aligned} \vartheta (t)&= \eta \sum _{j=1}^d \psi (t) \chi _j(t) {{\mathrm{sign}}}(R_{0,j}(t)) |R_{0,j}(t)|^{1/p} \big ( \Theta _\mu ^j \big )_\lambda , \\ w(t)&= \eta ^{-1} \sum _{j=1}^d \psi (t) \chi _j(t) |R_{0,j}(t)|^{1/p'} \big ( W_\mu ^j \big )_\lambda , \end{aligned} \end{aligned}$$

where, coherent with (18),

$$\begin{aligned} \big ( \Theta _\mu ^j \big )_\lambda (x) = \Theta _\mu ^j (\lambda x), \qquad \big ( W_\mu ^j \big )_\lambda (x) = W_\mu ^j (\lambda x). \end{aligned}$$

Notice that $\vartheta$ and *w* are smooth functions, thanks to the cutoffs $\chi _j$. Notice also that $\vartheta + \vartheta _c$ has zero mean value in $\mathbb {T}^d$ at each time *t*. To define $w_c$, notice first that

$$\begin{aligned} - \text {div }w(t) = -\, \eta ^{-1} \sum _{j=1}^d \nabla \Big ( \psi (t) \chi _j(t) \big |R_{0,j}(t)\big |^{1/p} \Big ) \cdot \big (W_\mu ^j \big )_\lambda \end{aligned}$$

is sum of terms of the form $f \cdot g_\lambda$, each term has zero mean value (being a divergence) and the fast oscillatory term $W_\mu ^j$ has zero mean value as well. We can therefore apply Lemma 2.3 and define

43 $$\begin{aligned} w_c(t) := -\, \eta ^{-1} \sum _{j=1}^d {\mathcal {R}} \bigg ( \nabla \Big ( \psi (t) \chi _j(t) \big |R_{0,j}(t)\big |^{1/p} \Big ) \cdot \big (W_\mu ^j \big )_\lambda \bigg ). \end{aligned}$$

Then $\text {div }(w + w_c) = 0$ and thus

$$\begin{aligned} \text {div }u_1 = \text {div }u_ 0 + \text {div }(w + w_c) = 0. \end{aligned}$$

Moreover, by Remark 2.5, $w_c$ is smooth in (*t*, *x*).

### Estimates on the Perturbation

In this section we provide some estimates on $\vartheta$, $\vartheta _c$, *w*, $w_c$.

#### Lemma 4.3

($L^p$-norm of $\vartheta$) For every time $t \in [0,1]$,

$$\begin{aligned} \Vert \vartheta (t)\Vert _{L^p(\mathbb {T}^d)} \le \frac{M}{2} \eta \Vert R_0(t)\Vert ^{1/p}_{L^1(\mathbb {T}^d)} + \frac{C(\eta , \delta , \Vert R_0(t)\Vert _{C^1(\mathbb {T}^d)})}{\lambda ^{1/p}}. \end{aligned}$$

#### Proof

The perturbation $\vartheta$ is the sum of functions of the form $f g_\lambda$. Therefore we can apply the improved Hölder inequality, Lemma 2.1, to get

$$\begin{aligned} \begin{aligned} \Vert \vartheta (t)\Vert _{L^p}&\le \eta \sum _{j=1}^d \bigg \Vert \psi (t)\chi _j(t) {{\mathrm{sign}}}\big ( R_{0,j}(t) \big ) \big |R_{0,j}(t)\big |^{1/p} \bigg \Vert _{L^p} \Vert \Theta _\mu ^j\Vert _{L^p} \\&\quad + \frac{C_p}{\lambda ^{1/p}} \Big \Vert \psi (t) \chi _j(t) {{\mathrm{sign}}}( R_{0,j}(t)) \big |R_{0,j}(t)\big |^{1/p} \Big \Vert _{C^1(\mathbb {T}^d)} \Vert \Theta _\mu ^j\Vert _{L^p}. \end{aligned} \end{aligned}$$

Notice now that

$$\begin{aligned} \bigg \Vert \psi (t) \chi _j(t) {{\mathrm{sign}}}\Big ( R_{0,j}(t) \Big ) \big |R_{0,j}(t)\big |^{1/p} \bigg \Vert _{L^p} \le \Big \Vert \big |R_{0,j}(t)\big |^{1/p} \Big \Vert _{L^p} \le \Vert R_0(t)\Vert _{L^1}^{1/p} \end{aligned}$$

and, recalling the definition of the cutoff $\chi _j$ in Sect. 4.2,

$$\begin{aligned} \Big \Vert \psi (t) \chi _j(t) {{\mathrm{sign}}}( R_{0,j}(t)) \big |R_{0,j}(t)\big |^{1/p} \Big \Vert _{C^1(\mathbb {T}^d)} \le C\big (\delta , \Vert R_0(t)\Vert _{C^1(\mathbb {T}^d)} \big ). \end{aligned}$$

Therefore, using the bounds on $\Vert \Theta _\mu ^j\Vert _{L^p}$ provided in (37), we get

$$\begin{aligned} \Vert \vartheta (t)\Vert _{L^p} \le \frac{M}{2}\eta \Vert R_0(t)\Vert _{L^1}^{1/p} + \frac{C(\eta , \delta , \Vert R_0(t)\Vert _{C^1})}{\lambda ^{1/p}}. \end{aligned}$$

$$\square $$

#### Lemma 4.4

(Estimate on $\vartheta _c$) It holds

$$\begin{aligned} |\vartheta _c(t)| \le \frac{C(\eta , \Vert R_0(t)\Vert _{C^1(\mathbb {T}^d)})}{\lambda }. \end{aligned}$$

#### Proof

We use Lemma 2.6:

$$\begin{aligned} \begin{aligned} |\vartheta _c(t)|&\le \eta \sum _{j=1}^d \frac{\sqrt{d} \Vert R_0(t)\Vert _{C^1(\mathbb {T}^d)} \Vert \Theta _\mu ^j\Vert _{L^1}}{\lambda } \\&\le \frac{C(\eta , \Vert R_0(t)\Vert _{C^1(\mathbb {T}^d)})}{\lambda }. \end{aligned} \end{aligned}$$

$$\square $$

#### Lemma 4.5

($L^{p'}$ norm of *w*) For every time $t \in [0,1]$,

$$\begin{aligned} \Vert w(t)\Vert _{L^{p'}(\mathbb {T}^d)} \le \frac{M}{2\eta } \Vert R_0(t)\Vert ^{1/p'}_{L^1(\mathbb {T}^d)} + \frac{C(\eta , \delta , \Vert R_0(t)\Vert _{C^1(\mathbb {T}^d)})}{\lambda ^{1/p'}}. \end{aligned}$$

#### Proof

The proof is completely analogous to the proof of Lemma 4.3, with $\eta ^{-1}$ instead of $\eta$ and $\Vert W_\mu ^j\Vert _{L^{p'}}$ instead of $\Vert \Theta _\mu ^j\Vert _{L^p}$, and thus it is omitted. $\square$

#### Lemma 4.6

($W^{1,{\tilde{p}}}$ norm of *w*) For every time $t \in [0,1]$,

$$\begin{aligned} \Vert w(t)\Vert _{W^{1, {\tilde{p}}}(\mathbb {T}^d)} \le C \Big (\eta , \Vert R_0\Vert _{C^1} \Big ) \lambda \mu ^{-\gamma }. \end{aligned}$$

#### Proof

We have

$$\begin{aligned} Dw(t,x) = \eta ^{-1} \psi (t) \sum _{j=1}^d W_\mu ^j (\lambda x) \otimes D \Big ( \chi _j |R_{0,j}|^{1/p'} \Big ) + \lambda \chi _j |R_{0,j}|^{1/p'} D W_\mu ^j(\lambda x), \end{aligned}$$

from which we get the pointwise estimate

$$\begin{aligned} |Dw(t,x)| \le C\big (\eta , \delta , \Vert R_0\Vert _{C^1} \big ) \sum _{j=1}^d \bigg (|W_\mu ^j(\lambda x)| + \lambda |D W_\mu ^j(\lambda x)| \bigg ). \end{aligned}$$

We can take now the $L^{{\tilde{p}}}$ norm of *Dw*(*t*) and use (38) to get

$$\begin{aligned} \begin{aligned} \Vert Dw(t)\Vert _{L^{{\tilde{p}}}}&\le C \big (\eta , \delta , \Vert R_0\Vert _{C^1} \big ) \sum _{j=1}^d \bigg ( \Vert W_\mu ^j\Vert _{L^{{\tilde{p}}}} + \lambda \Vert DW_\mu ^j\Vert _{L^{{\tilde{p}}}} \bigg ) \\&\le C \big (\eta , \delta , \Vert R_0\Vert _{C^1} \big ) \lambda \mu ^{-\gamma }. \end{aligned} \end{aligned}$$

A similar (and even easier) computation holds for $\Vert w(t)\Vert _{L^{{\tilde{p}}}}$, thus concluding the proof of the lemma. $\square$

#### Lemma 4.7

($L^{p'}$ norm of $w_c$) For every time $t \in [0,1]$,

$$\begin{aligned} \Vert w_c(t)\Vert _{L^{p'}(\mathbb {T}^d)} \le \frac{C (\eta , \delta , \Vert R_0\Vert _{C^2} )}{\lambda }. \end{aligned}$$

#### Proof

The corrector $w_c$ is defined in (43) using the antidivergence operator of Lemma 2.3. We can thus use the bounds given by that lemma, with $k=0$, to get

$$\begin{aligned} \begin{aligned} \Vert w_c(t)\Vert _{L^{p'}}&\le \eta ^{-1} \sum _{j=1}^d \frac{C_{0,p'}}{\lambda } \Big \Vert \nabla \big ( \psi (t) \chi _j(t) |R_{0,j}(t)|^{1/p'} \big ) \Big \Vert _{C^1(\mathbb {T}^d)} \big \Vert W_\mu ^j\big \Vert _{L^{p'}} \\&\le \frac{C(\eta , \delta , \Vert R_0\Vert _{C^2})}{\lambda } \sum _{j=1}^d \Vert W_\mu ^j\Vert _{L^{p'}} \\ \text {(by } (37))&\le \frac{C(\eta , \delta , \Vert R_0\Vert _{C^2})}{\lambda }. \end{aligned} \end{aligned}$$

$$\square $$

#### Lemma 4.8

($W^{1,{\tilde{p}}}$ norm of $w_c$) For every time $t \in [0,1]$,

$$\begin{aligned} \Vert w_c(t)\Vert _{W^{1, {\tilde{p}}}(\mathbb {T}^d)} \le C \Big (\eta , \delta , \Vert R_0\Vert _{C^3} \Big ) \mu ^{-\gamma }. \end{aligned}$$

#### Proof

We estimate only $\Vert Dw_c(t)\Vert _{L^{{\tilde{p}}}}$, the estimate for $\Vert w_c(t)\Vert _{L^{{\tilde{p}}}}$ is analogous and even easier. We use once again the bounds provided by Lemma 2.3 with $k=1$:

$$\begin{aligned} \begin{aligned} \Vert Dw_c(t)\Vert _{L^{{\tilde{p}}}}&\le \eta ^{-1} C_{1,{\tilde{p}}} \sum _{j=1}^d \Big \Vert \nabla \big ( \psi (t) \chi _j(t) |R_{0,j}(t)|^{1/p} \big ) \Big \Vert _{C^2(\mathbb {T}^d)} \big \Vert W_\mu ^j\big \Vert _{W^{1,{\tilde{p}}}} \\ \text {(by } (38))&\le C(\eta , \delta , \Vert R_0\Vert _{C^3}) \mu ^{-\gamma }. \end{aligned} \end{aligned}$$

$$\square $$

## The New Defect Field

In this section we continue the proof of Proposition 3.1, defining the new defect field $R_1$ and proving some estimates on it.

### Definition of the New Defect Field

We want to define $R_1$ so that

$$\begin{aligned} -\,\text {div }R_1 = \partial _t \rho _1 + \text {div }(\rho _1 u_1). \end{aligned}$$

Let us compute

44 $$\begin{aligned} \begin{aligned} \partial _t \rho _1 + \text {div }(\rho _1 u_1)&= \text {div }(\vartheta w - R_0) \\&\quad + \partial _t (\vartheta + \vartheta _c) + \text {div }( \vartheta u_0 + \rho _0 w ) \\&\quad + \text {div }(\rho _0 w_c + \vartheta _c u_0 + \vartheta w_c + \vartheta _c w + \vartheta _c w_c) \\&= \text {div }\Big [ (\vartheta w - R_0) \\&\quad \quad \qquad + \big (\nabla \Delta ^{-1} \partial _t (\vartheta + \vartheta _c) + \vartheta u_0 + \rho _0 w \big ) \\&\quad \quad \qquad + \big ( \rho _0 w_c + \vartheta _c u_0 + \vartheta w_c + \vartheta _c w + \vartheta _c w_c \big ) \Big ] \\&= \text {div }\Big [ (\vartheta w - R_0) + R^{\mathrm{linear}} + R^{\mathrm{corr}} \Big ] \\ \end{aligned} \end{aligned}$$

where we put

45 $$\begin{aligned} \begin{aligned} R^{\mathrm{linear}}&:= \nabla \Delta ^{-1} \partial _t ( \vartheta + \vartheta _c ) + \vartheta u_0 + \rho _0 w \\ R^{\mathrm{corr}}&:= \rho _0 w_c + \vartheta _c u_0 + \vartheta w_c + \vartheta _c w + \vartheta _c w_c . \end{aligned} \end{aligned}$$

Note that we can apply the antidivergence operator $\nabla \Delta ^{-1}$ to $\partial _t (\vartheta + \vartheta _c)$, since it has zero mean value. Let us now consider the term $\vartheta w - R_0$. Recall from Proposition 4.1, that, for $j \ne k$, $\mathrm {supp} \ \Theta _\mu ^j \cap \mathrm {supp} \ W_\mu ^k = \emptyset$. Coherent with (18), we use the notation

$$\begin{aligned} (\Theta _\mu ^j W_\mu ^j)_\lambda (x) = \Theta _\mu ^j(\lambda x) W_\mu ^j(\lambda x). \end{aligned}$$

We have

$$\begin{aligned} \begin{aligned} \vartheta (t) w(t) - R_0(t)&= \sum _{j=1}^d \psi ^2(t) \chi _j^2(t) R_{0,j}(t) (\Theta _\mu ^j W_\mu ^j)_\lambda - R_0(t)\\&= \sum _{j=1}^d \psi ^2(t) \chi _j^2(t) R_{0,j}(t) \big [(\Theta _\mu ^j W_\mu ^j)_\lambda - e_j \big ] \\&\quad + \psi ^2(t) \sum _{j=1}^d \big [ \chi _j^2(t) - 1 \big ] R_{0,j}(t) e_j \\&\quad + \big [ \psi ^2(t) - 1 \big ] R_0(t)\\&= \sum _{j=1}^d \psi ^2(t) \chi _j^2(t) R_{0,j}(t) \big [ (\Theta _\mu ^j W_\mu ^j)_\lambda - e_j \big ] \\&\quad + R^\chi (t) + R^\psi (t), \end{aligned} \end{aligned}$$

where we put

46 $$\begin{aligned} \begin{aligned} R^\chi (t)&:= \psi ^2(t) \sum _{j=1}^d \big [ \chi _j^2(t) - 1 \big ] R_{0,j}(t) e_j, \\ R^\psi (t)&:= \big [ \psi ^2(t) - 1 \big ] R_0(t). \end{aligned} \end{aligned}$$

Thus, using again Proposition 4.1, and in particular the fact that $\text {div }(\Theta _\mu ^j W_\mu ^j) = 0$, we get

47 $$\begin{aligned} \begin{aligned} \text {div }(\vartheta (t) w(t) - R_0(t))&= \sum _{j=1}^d \nabla \Big (\psi ^2(t) \chi _j^2(t) R_{0,j}(t) \Big ) \cdot \Big [ (\Theta _\mu ^j W_\mu ^j)_\lambda - e_j \Big ] \\&\quad + \text {div }(R^\chi + R^\psi ). \end{aligned} \end{aligned}$$

Each term in the summation over *j* has the form $f \cdot g_\lambda$ and it has zero mean value, being a divergence. Moreover, again by Proposition 4.1,Therefore we can apply Lemma 2.3 and define

48 $$\begin{aligned} R^{\mathrm{quadr}}(t) := \sum _{j=1}^d {\mathcal {R}} \bigg (\nabla \Big ( \psi ^2(t) \chi _j^2(t) R_{0,j}(t) \Big ) \cdot \Big [ (\Theta _\mu ^j W_\mu ^j)_\lambda - e_j \Big ] \bigg ). \end{aligned}$$

By Remark 2.5, $R^{\mathrm{quadr}}$ is smooth in (*t*, *x*). Summarizing, from (44) and (47) we get

$$\begin{aligned} \partial _t \rho _1 + \text {div }(\rho _1 u_1) = \text {div }\Big [R^{\mathrm{quadr}} + R^\chi + R^\psi + R^{\mathrm{linear}} + R^{\mathrm{corr}} \Big ] . \end{aligned}$$

We thus define

49 $$\begin{aligned} - R_1 : = R^{\mathrm{quadr}} + R^\chi + R^\psi + R^{\mathrm{linear}} + R^{\mathrm{corr}}. \end{aligned}$$

Aim of the next section will be to get an estimate in $L^1$ for $R_1(t)$, by estimating separately each term in (49).

### Estimates on the Defect Field

We now prove some estimates on the different terms which define $R_1$.

#### Lemma 5.1

(Estimate on $R^{\mathrm{quadr}}$) For every $t \in [0,1]$,

$$\begin{aligned} \Vert R^{\mathrm{quadr}}(t)\Vert _{L^1(\mathbb {T}^d)} \le \frac{C(\delta , \Vert R_0\Vert _{C^2})}{\lambda }. \end{aligned}$$

#### Proof

$R^{\mathrm{quadr}}$ is defined in (48) using Lemma 2.3. Observe first that

$$\begin{aligned} \big \Vert \nabla \big ( \psi ^2(t) \chi _j^2(t) R_{0,j}(t)\big )\big \Vert _{C^1(\mathbb {T}^d)} \le C(\delta , \Vert R_0\Vert _{C^2} ). \end{aligned}$$

Applying the bounds provided by Lemma 2.3, with $k=0$, and (37) we get

$$\begin{aligned} \begin{aligned} \Vert R^{\mathrm{quadr}}&(t)\Vert _{L^1(\mathbb {T}^d)} \\&\le \sum _{j=1}^d \frac{C_{0,1}}{\lambda } \big \Vert \nabla \big ( \psi ^2(t) \chi _j^2(t) R_{0,j}(t)\big )\big \Vert _{C^1} \big \Vert \Theta _\mu ^j W_\mu ^j - e_j\big \Vert _{L^1(\mathbb {T}^d)} \\&\le \sum _{j=1}^d \frac{C(\delta , \Vert R_0\Vert _{C^2})}{\lambda }. \end{aligned} \end{aligned}$$

$$\square $$

#### Lemma 5.2

(Estimate on $R^{\chi }$) For every $t \in [0,1]$

$$\begin{aligned} \Vert R^\chi (t)\Vert _{L^1(\mathbb {T}^d)} \le \frac{\delta }{2} \end{aligned}$$

#### Proof

Notice that $\chi _j(t,x) = 1$ if $|R_{0,j}(t,x)| \ge \delta /(2d)$. Therefore $R^\chi (t,x) \ne 0$ only when $|R_{0,j}(t,x)| \le \delta /(2d)$. We thus have the pointwise estimate

$$\begin{aligned} |R^\chi (t,x)| \le \sum _{j=1}^d |\chi _j(t,x)^2 - 1| |R_{0,j}(t,x)| \le \frac{\delta }{2}. \end{aligned}$$

from which the conclusion easily follows. $\square$

#### Lemma 5.3

(Estimate on $R^{\psi }$) It holds

$$\begin{aligned} \Vert R^\psi (t)\Vert _{L^1(\mathbb {T}^d)} \le {\left\{ \begin{array}{ll} 0, &{} t \in I_\sigma , \\ \Vert R_0(t)\Vert _{L^1(\mathbb {T}^d)}, &{} t \in [0,1] {\setminus } I_\sigma . \end{array}\right. } \end{aligned}$$

#### Proof

The proof follows immediately from the definition of $R^\psi$ in (46) and the definition of the cutoff $\psi$. $\square$

#### Lemma 5.4

(Estimate on $R^{\mathrm{linear}}$) For every $t \in [0,1]$

$$\begin{aligned} \Vert R^{\mathrm{linear}}(t)\Vert _{L^1(\mathbb {T}^d)} \le C\big (\eta , \delta , \sigma , \Vert \rho _0\Vert _{C^0}, \Vert u_0\Vert _{C^0}, \Vert R_0\Vert _{C^0}\big ) \mu ^{-\gamma }. \end{aligned}$$

#### Proof

At each time $t \in [0,1]$,

$$\begin{aligned} \begin{aligned} \Vert R^{\mathrm{linear}}&(t)\Vert _{L^1(\mathbb {T}^d)} \\&\le \Vert \nabla \Delta ^{-1} \partial _t ( \vartheta (t) + \vartheta _c(t) )\Vert _{L^1(\mathbb {T}^d)} + \Vert \vartheta (t) u_0(t)\Vert _{L^1(\mathbb {T}^d)} + \Vert \rho _0(t) w(t)\Vert _{L^1(\mathbb {T}^d)} \\&\le \Vert \partial _t \vartheta (t)\Vert _{L^1(\mathbb {T}^d)} + |\vartheta _c'(t)| + \Vert \vartheta (t) u_0(t)\Vert _{L^1(\mathbb {T}^d)} + \Vert \rho _0(t) w(t)\Vert _{L^1(\mathbb {T}^d)}, \end{aligned} \end{aligned}$$

where the first term was estimated using Lemma 2.2. We now separately estimate each term in the last sum.

*1. Estimate on* $\Vert \partial _t \vartheta (t)\Vert _{L^1}$. We have

$$\begin{aligned} \partial _t \vartheta (t) = \eta \sum _{j=1}^d \partial _t \Big ( \psi (t) \chi _j(t,x) {{\mathrm{sign}}}(R_{0,j}(t,x)) |R_{0,j}(t,x)|^{1/p} \Big ) \Theta _\mu ^j(\lambda x) \end{aligned}$$

from which we get the pointwise estimate

$$\begin{aligned} |\partial _t \vartheta (t)| \le C(\eta , \delta , \sigma , \Vert R_0\Vert _{C^1}) \sum _{j=1}^d |\Theta _\mu ^j(\lambda x)|. \end{aligned}$$

Using (38), we deduce

$$\begin{aligned} \Vert \partial _t \vartheta (t)\Vert _{L^1} \le C(\eta , \delta , \sigma , \Vert R_0\Vert _{C^1}) \mu ^{-\gamma }. \end{aligned}$$

*2. Estimate on* $|\vartheta _c'(t)|$. We have

$$\begin{aligned} |\vartheta _c'(t)| \le \Vert \partial _t \vartheta (t)\Vert _{L^1} \le C(\eta , \delta , \sigma , \Vert R_0\Vert _{C^1}) \mu ^{-\gamma }. \end{aligned}$$

*3. Estimate on* $\Vert \vartheta (t) u_0(t)\Vert _{L^1}$. We now use the classical Hölder inequality to estimate

$$\begin{aligned} \begin{aligned} \Vert \vartheta (t) u_0(t)\Vert _{L^1}&\le \Vert u_0\Vert _{C^0} \Vert \vartheta (t)\Vert _{L^1} \\&\le \eta \Vert u_0\Vert _{C^0} \sum _{j=1}^d \big \Vert |R_{0,j}|^{1/p} \big \Vert _{C^0} \Vert \Theta _\mu ^j\Vert _{L^1} \\ \text {(by } (38))&\le C\big (\eta , \Vert u_0\Vert _{C^0}, \Vert R_0\Vert _{C^0}\big ) \mu ^{-\gamma }. \end{aligned} \end{aligned}$$

*4. Estimate on* $\Vert \rho _0(t) w(t)\Vert _{L^1}$. Similarly, again using the classical Hölder inequality,

$$\begin{aligned} \begin{aligned} \Vert \rho _0(t) w(t)\Vert _{L^1}&\le \Vert \rho _0\Vert _{C^0} \Vert w(t)\Vert _{L^1} \\&\le \eta ^{-1} \Vert \rho _0\Vert _{C^0} \sum _{j=1}^d \big \Vert |R_{0,j}|^{1/p'} \big \Vert _{C^0} \Vert W_\mu ^j\Vert _{L^1} \\ \text {(by } (38))&\le C\big (\eta , \Vert \rho _0\Vert _{C^0}, \Vert R_0\Vert _{C^0}\big ) \mu ^{-\gamma }. \end{aligned} \end{aligned}$$

$$\square $$

#### Lemma 5.5

(Estimate on $R^{\mathrm{corr}}$) For every $t \in [0,1]$,

$$\begin{aligned} \Vert R^{\mathrm{corr}}(t)\Vert _{L^1(\mathbb {T}^d)} \le \frac{C(\eta , \delta , \Vert \rho _0\Vert _{C^0}, \Vert u_0\Vert _{C^0}, \Vert R_0\Vert _{C^2})}{\lambda }. \end{aligned}$$

#### Proof

We estimate separately each term in the definition (45) of $R^{\mathrm{corr}}$.

*1. Estimate on* $\rho _0 w_c$. By the classical Hölder inequality,

$$\begin{aligned} \begin{aligned} \Vert \rho _0(t) w_c(t)\Vert _{L^1}&\le \Vert \rho _0\Vert _{C^0} \Vert w_c(t)\Vert _{L^1} \\&\le \Vert \rho _0\Vert _{C^0} \Vert w_c(t)\Vert _{L^{p'}} \\ \text {(by Lemma } 4.7)&\le \frac{C(\eta , \delta , \Vert \rho _0\Vert _{C^0}, \Vert R_0\Vert _{C^2}) }{\lambda }. \end{aligned} \end{aligned}$$

*2. Estimate on* $\vartheta _c u_0$. We use Lemma 4.4:

$$\begin{aligned} \begin{aligned} \Vert \vartheta _c(t) u_0(t)\Vert _{L^1} \le |\vartheta _c(t)| \Vert u_0\Vert _{C^0} \le \frac{C(\eta , \Vert u_0\Vert _{C^0}, \Vert R_0\Vert _{C^2})}{\lambda }. \end{aligned} \end{aligned}$$

*3. Estimate on* $\vartheta w_c$. We use Lemmas 4.3 and  4.7:

$$\begin{aligned} \begin{aligned} \Vert \vartheta (t) w_c(t)\Vert _{L^1} \le \Vert \vartheta (t)\Vert _{L^p} \Vert w_c(t)\Vert _{L^{p'}} \le \frac{C(\eta , \delta , \Vert R_0\Vert _{C^2})}{\lambda }. \end{aligned} \end{aligned}$$

*4. Estimate on* $\vartheta _c w$. We use Lemmas 4.4 and  4.5:

$$\begin{aligned} \begin{aligned} \Vert \vartheta _c(t) w(t)\Vert _{L^1}&\le |\vartheta _c(t)| \Vert w(t)\Vert _{L^1} \\&\le |\vartheta _c(t)| \Vert w(t)\Vert _{L^{p'}} \\&\le \frac{C(\eta , \delta , \Vert R_0\Vert _{C^2})}{\lambda }. \end{aligned} \end{aligned}$$

*5. Estimate on* $\vartheta _c w_c$. We use Lemmas 4.4 and  4.7:

$$\begin{aligned} \begin{aligned} \Vert \vartheta _c(t) w_c(t)\Vert _{L^1}&\le |\vartheta _c(t)| \Vert w_c(t)\Vert _{L^1} \\&\le |\vartheta _c(t)| \Vert w_c(t)\Vert _{L^{p'}} \\&\le \frac{C(\eta , \delta , \Vert R_0\Vert _{C^2})}{\lambda ^2}. \end{aligned} \end{aligned}$$

$$\square $$

#### Remark 5.6

In estimating $R^{\mathrm{corr}}$ the only term where we really need the fast oscillation $\lambda$ is the estimate on $\vartheta w_c$. All the other terms could be alternatively estimated using the concentration parameter $\mu$, since, by (38), $|\vartheta _c(t)|, \Vert w_c(t)\Vert _{L^1}, \Vert w(t)\Vert _{L^1} \le \text {const. } \ \mu ^{-\gamma }$. In this way we would obtained the less refined estimate

$$\begin{aligned} \Vert R^{\mathrm{corr}}(t)\Vert _{L^1} \le \frac{C}{\lambda } + \frac{C}{\mu ^\gamma }, \end{aligned}$$

which is however enough to prove Proposition 3.1.

## Proof of Proposition 3.1

In this section we conclude the proof of Proposition 3.1, proving estimates (26a)–(26d). We will choose

$$\begin{aligned}\mu = \lambda ^c\end{aligned}$$

for a suitable $c>1$ and $\lambda$ sufficiently large.

*1. Estimate* (26a). We have

$$\begin{aligned} \begin{aligned} \Vert \rho _1(t) - \rho _0(t)\Vert _{L^p}&\le \Vert \vartheta _0(t)\Vert _{L^p} + |\vartheta _c(t)| \\ \text {(Lemmas } 4.3 \hbox { and } 4.4)&\le \frac{M}{2} \eta \Vert R_0(t)\Vert ^{1/p}_{L^1} + \frac{C(\eta , \delta , \Vert R_0(t)\Vert _{C^1})}{\lambda ^{1/p}} \\&\qquad + \frac{C(\eta , \Vert R_0(t)\Vert _{C^1})}{\lambda } \\&\le M \eta \Vert R_0(t)\Vert ^{1/p}_{L^1}, \end{aligned} \end{aligned}$$

if the constant $\lambda$ is chosen large enough. Notice also that, if $t \in [0,1] {\setminus } I_{\sigma /2}$, then $\vartheta (t) \equiv 0$ and $\vartheta _c(t) = 0$, thanks to the cutoff $\psi$ in (42). Therefore (26a) is proven.

*2. Estimate* (26b). The estimate uses Lemmas 4.5 and 4.7 and it is completely similar to what we just did for (26a).

*4. Estimate* (26c). By Lemma 4.6,

$$\begin{aligned} \Vert w(t)\Vert _{W^{1, {\tilde{p}}}} \le C \Big (\eta , \Vert R_0\Vert _{C^1} \Big ) \lambda \mu ^{-\gamma } \le \delta , \end{aligned}$$

if $\mu$ is chosen of the form $\mu = \lambda ^c$ with $c > 1/\gamma$ and $\lambda$ is chosen large enough.

*4. Estimate* (26d). Recall the definition of $R_1$ in (49). Using Lemmas 5.1,  5.2, 5.3, 5.4, 5.5, for $t \in I_\sigma$, we get

$$\begin{aligned} \begin{aligned} \Vert R_1(t)\Vert _{L^1}&\le \frac{\delta }{2} + C(\eta , \delta , \sigma , \Vert \rho _0\Vert _{C^0}, \Vert u_0\Vert _{C^0}, \Vert R_0\Vert _{C^2}) \bigg ( \frac{1}{\lambda } + \frac{1}{\mu ^\gamma } \bigg ) \\&\le \frac{\delta }{2} + C(\eta , \delta , \sigma , \Vert \rho _0\Vert _{C^0}, \Vert u_0\Vert _{C^0}, \Vert R_0\Vert _{C^2}) \bigg ( \frac{1}{\lambda } + \frac{1}{\lambda ^{c \gamma }} \bigg ) \\&\le \delta \end{aligned} \end{aligned}$$

provided $\lambda$ is chosen large enough. Similarly, for $t \in I_{\sigma /2} {\setminus } I_\sigma$, we have

$$\begin{aligned} \begin{aligned} \Vert&R_1(t)\Vert _{L^1} \\&\le \Vert R_0(t)\Vert _{L^1} + \frac{\delta }{2} + C(\eta , \delta , \sigma , \Vert \rho _0\Vert _{C^0}, \Vert u_0\Vert _{C^0}, \Vert R_0\Vert _{C^2}) \bigg ( \frac{1}{\lambda } + \frac{1}{\mu ^{\gamma }} \bigg ) \\&\le \Vert R_0(t)\Vert _{L^1} + \frac{\delta }{2} + C(\eta , \delta , \sigma , \Vert \rho _0\Vert _{C^0}, \Vert u_0\Vert _{C^0}, \Vert R_0\Vert _{C^2}) \bigg ( \frac{1}{\lambda } + \frac{1}{\lambda ^{c \gamma }} \bigg ) \\&\le \Vert R_0(t)\Vert _{L^1} + \delta \end{aligned} \end{aligned}$$

if $\lambda$ is chosen large enough. Finally, for $t \in [0,1] {\setminus } I_{\sigma /2}$, the cutoff function $\psi (t) \equiv 0$, and thus $\vartheta (t) = \vartheta _c(t) = w(t) = w_c(t) = 0$. Therefore $R_1(t) = R_0(t)$.

## Sketch of the Proofs of Theorems 1.6, 1.9, 1.10

Theorems 1.6, 1.9, 1.10 can be proven in a very similar way to Theorem 1.2 and thus we present just a sketch of their proofs.

The proof of Theorem 1.2 follows from Proposition 3.1: similarly, for each one of Theorems 1.6, 1.9, 1.10 there is a corresponding *main* proposition, from which the proof the theorem follows.

### Sketch of the proof of Theorem 1.9

The proof of Theorem 1.9 follows from the next proposition, in a very similar way as Theorem 1.2 follows from Proposition 3.1. Let us consider the equation

50 $$\begin{aligned} \left\{ \begin{aligned}&\partial _t \rho + \text {div }(\rho u) -\Delta \rho = -\, \text {div }R, \\&\text {div }u = 0. \end{aligned} \right. \end{aligned}$$

#### Proposition 7.1

Proposition 3.1 holds with (50) instead of (25).

#### Sketch of the proof of Proposition 7.1

Exactly as in the proof of Proposition 3.1, we define the Mikado densities and fields as in Proposition 4.1 and we choose the exponents *a*, *b* as in Remark 4.2. We observe that, in addition to (37), (38), it also holds

51 $$\begin{aligned} \Vert \nabla \Theta _\mu ^j\Vert _{L^1} \le M \mu ^{-\gamma _4} \le M \mu ^{-\gamma } \end{aligned}$$

for

$$\begin{aligned} \gamma = \min \big \{\gamma _1, \gamma _2, \gamma _3, \gamma _4 \big \} > 0, \end{aligned}$$

where $\gamma _1, \gamma _2, \gamma _3$ were defined in Remark 4.2 and

$$\begin{aligned} \gamma _4 := \frac{d-1}{p'} - 1 > 0, \end{aligned}$$

because of the second condition in (15). Then the perturbations $\vartheta , w, \vartheta _c, w_c$ can be defined as in Sect. 4.2 and the estimates in Sect. 4.3 continue to hold. In the definition of the new defect field in Sect. 5 we want to define $R_1$ so that

$$\begin{aligned} -\text {div }R_1=\partial _t\rho _1+\text {div }(\rho _1u_1)-\Delta \rho _1, \end{aligned}$$

which leads to an additional term $\nabla \vartheta$ in the expression for $R^{\mathrm{linear}}$ in (45). As a consequence the only estimate which changes is Lemma 5.4. From (51) and the expression for $\vartheta$ in (42) we easily obtain

$$\begin{aligned} \Vert \nabla \theta \Vert _{L^1(\mathbb {T}^d)}\le C(\eta ,\delta ,\Vert R_0\Vert _{C^1})\lambda \mu ^{-\gamma }. \end{aligned}$$

Since we choose $\mu =\lambda ^c$ with $c>1/\gamma$ in Sect. 6, the final estimates for $\Vert R_1(t)\Vert _{L^1}$ continue to hold. This concludes the proof of proposition (and thence also the proof of Theorem 1.9). $\square$

### Sketch of the proof of Theorem 1.6

Also for Theorem 1.6 there is a *main* proposition, analog to Proposition 3.1.

#### Proposition 7.2

There exists a constant $M>0$ and an exponent $s \in (1, \infty )$ such that the following holds. Let $\eta , \delta , \sigma >0$ and let $(\rho _0, u_0, R_0)$ be a smooth solution of the continuity-defect equation (25). Then there exists another smooth solution $(\rho _1, u_1, R_1)$ of (25) such that

52a $$\begin{aligned} \Vert \rho _1(t) - \rho _0(t)\Vert _{L^s(\mathbb {T}^d)}&\le {\left\{ \begin{array}{ll} M \eta \Vert R_0(t)\Vert ^{1/s}_{L^1(\mathbb {T}^d)}, &{} t \in I_{\sigma /2}, \\ 0, &{} t \in [0,1] {\setminus } I_{\sigma /2}, \end{array}\right. } \end{aligned}$$

52b $$\begin{aligned} \Vert u_1(t) - u_0(t)\Vert _{L^{s'}(\mathbb {T}^d)}&\le {\left\{ \begin{array}{ll} M \eta ^{-1} \Vert R_0(t)\Vert ^{1/s'}_{L^1(\mathbb {T}^d)}, &{} t \in I_{\sigma /2}, \\ 0, &{} t \in [0,1] {\setminus } I_{\sigma /2}, \end{array}\right. } \end{aligned}$$

52c $$\begin{aligned} \left. \begin{aligned} \Vert \rho _1 (t) - \rho _0(t)\Vert _{W^{m,p}(\mathbb {T}^d)} \\ \Vert u_1(t) - u_0(t)\Vert _{W^{{\tilde{m}},{\tilde{p}}}(\mathbb {T}^d)} \end{aligned} \right\}&\le \ \ \delta , \end{aligned}$$

52d $$\begin{aligned} \Vert R_1(t)\Vert _{L^1(\mathbb {T}^d)}&\le {\left\{ \begin{array}{ll} \delta , &{} t \in I_\sigma , \\ \Vert R_0(t)\Vert _{L^1(\mathbb {T}^d)} + \delta , &{} t \in I_{\sigma /2} {\setminus } I_\sigma , \\ \Vert R_0(t)\Vert _{L^1(\mathbb {T}^d)}, &{} t \in [0,1] {\setminus } I_{\sigma /2}. \end{array}\right. } \end{aligned}$$

Theorem 1.6 can be deduced from Proposition 7.2 exactly in the same way as Theorem 1.2 was deduced from Proposition 3.1. The only difference here is the following. In general, it is not true that $\rho (t) \in L^p(\mathbb {T}^d)$, $u(t) \in L^{p'} (\mathbb {T}^d)$. Therefore the fact that $\rho u \in C((0,T); L^1(\mathbb {T}^d))$ is proven by showing that $\rho \in C((0,T); L^s(\mathbb {T}^d))$ (thanks to (52a)) and $u \in C((0,T); L^{s'} (\mathbb {T}^d))$ (thanks to (52b)).

#### Sketch of the proof of Proposition 7.2

The proof is analog to the proof of Proposition 3.1 presented in Sects. 4–6. Here, however, we need modify the “rate of concentration” of the Mikado fields defined in (39) to achieve better estimates on the derivatives. In other words, we have to modify the choice of *a*, *b* in (39), as follows. In order to get estimates (52a)–(52b), we want

53 $$\begin{aligned} \begin{aligned} \Vert \Theta _\mu ^j\Vert _{L^s(\mathbb {T}^d)}, \ \Vert W_\mu ^j\Vert _{L^{s'}(\mathbb {T}^d)},&\le \text {const.}, \end{aligned} \end{aligned}$$

to get (52c) we want

54 $$\begin{aligned} \begin{aligned} \Vert \Theta _\mu ^j\Vert _{W^{m, p}} \le \text { const } \cdot \mu ^{-\gamma }, \ \Vert W_\mu ^j\Vert _{W^{{\tilde{m}}, {\tilde{p}}}} \le \text { const } \cdot \mu ^{-\gamma }; \end{aligned} \end{aligned}$$

we also require

55 $$\begin{aligned} \begin{aligned} \Vert \Theta _\mu ^j\Vert _{L^1(\mathbb {T}^d)}, \le \text { const } \cdot \mu ^{-\gamma }, \ \ \Vert W_\mu ^j\Vert _{L^{1}(\mathbb {T}^d)} \le \text {const } \cdot \mu ^{-\gamma }, \ \ \end{aligned} \end{aligned}$$

for some positive constant $\gamma >0$. Compare (53) with the first and the second estimates in (37), compare (54) with the last estimate in (38) and compare (55) with the first and the second estimates in (38).

We want to find

56 $$\begin{aligned} a,b \in (0, d-1), \end{aligned}$$

so that $a+b = d-1$ and (54) is achieved. If we can do that, then condition (55) is a consequence of (35) and (56). Similarly, condition (53) is automatically satisfied, choosing

$$\begin{aligned} s = \frac{d-1}{a}, \quad s' = \frac{d-1}{b}. \end{aligned}$$

and observing that (56) implies $s,s' \in (1,\infty )$.

Using (35), we see that, to achieve (54), we need 
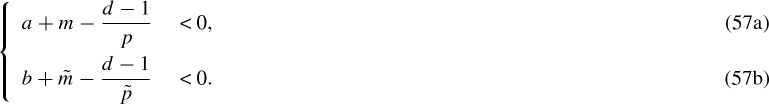
 Notice that, since $a+b = d-1$, (57b) is equivalent to

$$\begin{aligned} a > (d-1) \bigg ( 1 - \frac{1}{{\tilde{p}}} \bigg ) + {\tilde{m}}. \end{aligned}$$

It is then possible to find *a*, *b* satisfying (56) and (57), with $a+d = d-1$, if and only if

58 $$\begin{aligned} \max \Bigg \{ 0, \ (d-1) \bigg ( 1 - \frac{1}{{\tilde{p}}} \bigg ) + {\tilde{m}} \Bigg \} < \min \Bigg \{d-1, \ \frac{d-1}{p} - m \Bigg \} \end{aligned}$$

and this last condition is equivalent to (14).

Proposition 7.2 can now be proven exactly as we proved Proposition 3.1 in Sects. 4–6, this time using (53)–(55) instead of (37)–(38). $\square$

### Sketch of the proof of Theorem 1.10

Once again, also for Theorem 1.10 there is a *main* proposition, from which the proof of the theorem follows. Let us consider the equation

59 $$\begin{aligned} \left\{ \begin{aligned}&\partial _t \rho + \text {div }(\rho u) - L \rho = -\, \text {div }R, \\&\text {div }u = 0. \end{aligned} \right. \end{aligned}$$

Recall that *L* is a constant coefficient differential operator of order $k \in \mathbb {N}$, $k \ge 2$.

#### Proposition 7.3

Proposition 7.2 holds with (59) instead of (25).

#### Sketch of the proof of Proposition 7.3

Similarly to the proof of Proposition 7.2, we want to choose the exponents $a,b \in (0, d-1)$ in Proposition 4.1 so that (53)–(55) are satisfied and, moreover,

60 $$\begin{aligned} \Vert D^{k-1} \Theta _\mu ^j\Vert _{L^1(\mathbb {T}^d)} \le \text {const} \cdot \mu ^{-\gamma }, \end{aligned}$$

for some positive constant $\gamma >0$. As in Proposition 7.2, to get (53)–(55) we need (58). Moreover, condition (60) is satisfied, provided

$$\begin{aligned} a + (k-1) - (d-1) < 0, \end{aligned}$$

or, equivalently,

61 $$\begin{aligned} a < d - k. \end{aligned}$$

Putting together (58) and (61), we obtain the condition

$$\begin{aligned} \max \Bigg \{ 0, \ (d-1) \bigg ( 1 - \frac{1}{{\tilde{p}}} \bigg ) + {\tilde{m}} \Bigg \} < \min \Bigg \{d-1, \ d-k, \ \frac{d-1}{p} - m \Bigg \}. \end{aligned}$$

It is now not difficult to see that the last inequality is satisfied if and only if (17) holds.

Then the perturbations $\vartheta , w, \vartheta _c, w_c$ can be defined as in Sect. 4.2 and the estimates on the perturbations can be proven as in Proposition 7.2. In the definition of the new defect field we want to define $R_1$ so that

$$\begin{aligned} -\text {div }R_1=\partial _t\rho _1+\text {div }(\rho _1u_1)-L\rho _1. \end{aligned}$$

We can write $L = \text {div }{\tilde{L}}$, where ${\tilde{L}}$ is a constant coefficient differential operator of order $k-1$. This leads to an additional term ${\tilde{L}} \vartheta$ in the expression for $R^\mathrm{linear}$ (compare with (45)), which can be estimated using (60):

$$\begin{aligned} \Vert {\tilde{L}} \vartheta \Vert _{L^1(\mathbb {T}^d)}\le C(\eta ,\delta ,\Vert R_0\Vert _{C^1})\lambda ^{k-1} \mu ^{-\gamma }. \end{aligned}$$

Choosing $\mu =\lambda ^c$ with $c>(k-1)/\gamma$, we get the estimates for $\Vert R_1(t)\Vert _{L^1}$. This concludes the proof of the proposition (and thence also the proof of Theorem 1.10). $\square$

## References

[1] Aizenman M (1978). On vector fields as generators of flows: a counterexample to Nelson’s conjecture. Ann. Math..

[2] Alberti G, Bianchini S, Crippa G (2014). A uniqueness result for the continuity equation in two dimensions. J. Eur. Math. Soc..

[3] Alberti G, Crippa G, Mazzucato AL (2014). Exponential self-similar mixing and loss of regularity for continuity equations. C. R. Math. Acad. Sci. Paris.

[4] Alberti, G., Crippa, G., Mazzucato, A. L.: Exponential self-similar mixing by incompressible flows. arXiv:1605.02090 (2016)

[5] Ambrosio L (2004). Transport equation and Cauchy problem for BV vector fields. Invent. Math..

[6] Ambrosio L (2017). Well posedness of ODEs and continuity equations with nonsmooth vector fields, and applications. Rev. Mat. Comput..

[7] Ambrosio L, Colombo M, Figalli A (2015). Existence and uniqueness of maximal regular flows for non-smooth vector fields. Arch. Rational Mech. Anal..

[8] Bianchini, S., Bonicatto, P. A uniqueness result for the decomposition of vector fields in Rd. SISSA (2017)

[9] Bianchini S, Colombo M, Crippa G, Spinolo LV (2017). Optimality of integrability estimates for advection-diffusion equations. Nonlinear Differ. Equ. Appl. NoDEA.

[10] Buckmaster T (2015). Onsager’s conjecture almost everywhere in time. Commun. Math. Phys..

[11] Buckmaster T, De Lellis C, Isett P, Székelyhidi L (2015). Anomalous dissipation for 1/5-Hölder Euler flows. Ann. Math..

[12] Buckmaster T, De Lellis C, Székelyhidi L (2016). Dissipative Euler flows with Onsager-critical spatial regularity. Commun. Pure Appl. Math..

[13] Buckmaster, T., De Lellis, C., Székelyhidi Jr, L., Vicol, V.: Onsager’s conjecture for admissible weak solutions. arXiv:1701.08678 (2017)

[14] Buckmaster, T., Vicol, V.: Nonuniqueness of weak solutions to the Navier–Stokes equation. arXiv:1709.10033 (2017)

[15] Caravenna L, Crippa G (2016). Uniqueness and Lagrangianity for solutions with lack of integrability of the continuity equation. C. R. Math. Acad. Sci. Paris.

[16] Colombini F, Lerner N (2002). Uniqueness of continuous solutions for BV vector fields. Duke Math. J..

[17] Colombini, F., Luo, T., Rauch, J.: Uniqueness and nonuniqueness for nonsmooth divergence free transport. In: Seminaire: Équations aux Dérivées Partielles, 2002–2003. École Polytech., Palaiseau, 2003, pp. Exp. No. XXII–21

[18] Crippa G, Gusev N, Spirito S, Wiedemann E (2015). Non-uniqueness and prescribed energy for the continuity equation. Comm. Math. Sci..

[19] Crippa G, Spirito S (2015). Renormalized solutions of the 2D Euler equations. Commun. Math. Phys..

[20] Daneri S, Székelyhidi L (2017). Non-uniqueness and h-principle for Hölder-continuous weak solutions of the Euler equations. Arch. Rational Mech. Anal..

[21] De Lellis C, Székelyhidi L (2009). The Euler equations as a differential inclusion. Ann. Math..

[22] De Lellis C, Székelyhidi L (2012). Dissipative continuous Euler flows. Invent. Math..

[23] De Lellis C, Székelyhidi L (2014). Dissipative Euler flows and Onsager’s conjecture. J. Eur. Math. Soc..

[24] Depauw N (2003). Non unicité des solutions bornées pour un champ de vecteurs BV en dehors d’un hyperplan. C. R. Math. Acad. Sci. Paris.

[25] DiPerna RJ, Lions P-L (1989). On the Cauchy problem for Boltzmann equations: global existence and weak stability. Ann. Math..

[26] DiPerna RJ, Lions P-L (1989). Ordinary differential equations, transport theory and Sobolev spaces. Invent. Math..

[27] Frisch U (1995). Turbulence.

[28] Frisch U, Sulem P-L, Nelkin M (1978). A simple dynamical model of intermittent fully developed turbulence. J. Fluid Mech..

[29] Isett P (2016). A Proof of Onsager’s Conjecture. Ann. Math..

[30] Isett P, Vicol V (2015). Hölder continuous solutions of active scalar equations. Ann. PDE.

[31] Ladyženskaja, O. A., Solonnikov, V., Ural’ceva, N.: Linear and quasilinear equations of parabolic type. Vol. 23, Series in Translations of Mathematical Monographs. American Mathematical Society, 648pp (1968)

[32] Le Bris C, Lions P-L (2004). Renormalized solutions of some transport equations with partially $$W^{1,1}$$ velocities and applications. Annali di Matematica Pura ed Applicata.

[33] Le Bris C, Lions P-L (2008). Existence and uniqueness of solutions to Fokker–Planck type equations with irregular coefficients. Commun. Partial Differ. Equ..

[34] Lévy G (2016). On uniqueness for a rough transport-diffusion equation. C. R. Math. Acad. Sci. Paris.

[35] Lions P-L (1996). Mathematical Topics in Fluid Mechanics: Volume 1: Incompressible Models.

[36] Lions, P.-L.: Mathematical topics in fluid mechanics. Vol. 2, vol. 10 of Oxford Lecture Series in Mathematics and Its Applications. The Clarendon Press, New York (1998)

[37] Yao Y, Zlato A (2017). Mixing and un-mixing by incompressible flows. J. Eur. Math. Soc..

